# Melt electrowritten poly-lactic acid /nanodiamond scaffolds towards wound-healing patches

**DOI:** 10.1016/j.mtbio.2024.101112

**Published:** 2024-05-31

**Authors:** Xixi Wu, Wenjian Li, Lara Herlah, Marcus Koch, Hui Wang, Romana Schirhagl, Małgorzata K. Włodarczyk-Biegun

**Affiliations:** a Department of Biomedical Engineering, University Medical Centre, Ant. Deusinglaan 1, 9713, AW, Groningen, the Netherlands; b Polymer Science, Zernike Institute for Advanced Materials, Faculty of Science and Engineering, University of Groningen, Nijenborgh 4, 9747, AG, the Netherlands; c Advanced Production Engineering, Engineering and Technology Institute of Groningen, Faculty of Science and Engineering, University of Groningen, Nijenborgh 4, 9747, AG, the Netherlands; d INM – Leibniz Institute for New Materials, Campus D2 2, 66123, Saarbrücken, Germany; e Nanostructured Materials and Interfaces, Zernike Institute for Advanced Materials, Faculty of Science and Engineering, University of Groningen, Nijenborgh 4, 9747, AG, the Netherlands; f Biotechnology Centre, The Silesian University of Technology, Krzywoustego 8, 44-100, Gliwice, Poland

**Keywords:** Melt electrowriting, Nanodiamonds, Quaternized β-chitin, Wound healing

## Abstract

Multifunctional wound dressings, enriched with biologically active agents for preventing or treating infections and promoting wound healing, along with cell delivery capability, are highly needed. To address this issue, composite scaffolds with potential in wound dressing applications were fabricated in this study. The poly-lactic acid/nanodiamonds (PLA/ND) scaffolds were first printed using melt electrowriting (MEW) and then coated with quaternized β-chitin (QβC). The NDs were well-dispersed in the printed filaments and worked as fillers and bioactive additions to PLA material. Additionally, they improved coating effectiveness due to the interaction between their negative charges (from NDs) and positive charges (from QβC). NDs not only increased the thermal stability of PLA but also benefitted cellular behavior and inhibited the growth of bacteria. Scaffolds coated with QβC increased the effect of bacteria growth inhibition and facilitated the proliferation of human dermal fibroblasts. Additionally, we have observed rapid extracellular matrix (ECM) remodeling on QβC-coated PLA/NDs scaffolds. The scaffolds provided support for cell adhesion and could serve as a valuable tool for delivering cells to chronic wound sites. The proposed PLA/ND scaffold coated with QβC holds great potential for achieving fast healing in various types of wounds.

## Highlights

• The thermal stability and biological performance of poly-lactic acid (PLA) were enhanced by nanodiamonds (NDs).
• High-resolution PLA/ND scaffolds were for the first time printed using melt electrowriting (MEW).
• Quaternized β-chitin coated on PLA/ND synergistically improved cell proliferation and bacteria inhibition.
• Rapid extracellular matrix (ECM) remodeling on QBC-coated PLA/NDs scaffolds was observed.
• PLA/ND scaffold coated with QBC holds great potential for wound dressings.

## Graphical abstract

**Figure:**
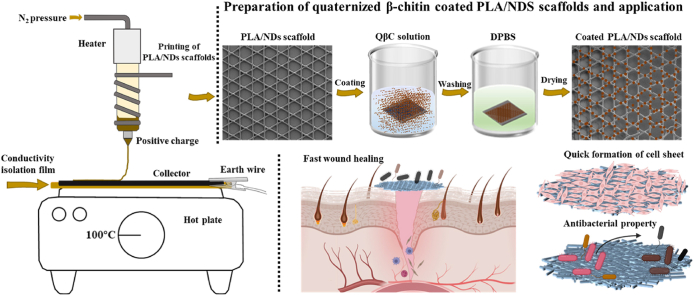

## 1 Introduction

Wounds can appear due to acute traumas, surgeries, burns, or chronic metabolic diseases [1]. Medical dressings applied to wound surfaces protect wounds from contamination and promote healing [2], to prevent microbial infection and facilitate the wound healing process by adjusting the cell functions. Wound dressings can include biologically active molecules like growth factors or nanoparticles, for example, vascular endothelial growth factor (VEGF), nanodiamonds, or silver nanoparticles [[3], [4], [5]]. During wound healing, dermal fibroblasts are critical to prevent inflammation, their proliferation plays a key role in the deposition and remodeling of extracellular matrix (ECM), and wound contraction [6]. Multiple studies showed that dermal fibroblasts in chronic non-healing wounds display abnormal phenotypes, including decreased proliferation, early senescence, downregulation of cytokine release, and diminished response to growth factors [7,8]. Hence, the use of advanced dressing materials without competent cells has failed to robustly improve the healing process of chronic wounds.

An effective approach to cure chronic wounds is delivering viable cells to the injury sites, including stem cells [9,10], fibroblasts [11,12] or fibroblasts with keratinocytes [13]. These cells provide growth factors and cytokines to support the function of the patient's cells [14]. Several methods have been developed to deliver cells into skin wounds such as invasive injection. However, the approach suffered from a limited retention time and required multiple delivery rounds [9,15]. Therefore, the use of scaffolds was proposed. Yoshikawa et al. [16] suggested using a collagen sponge with embedded mesenchymal stem/stromal cells (MSC). The use of the sponge demonstrated higher efficacy and less invasiveness than the direct injection of MSCs in the wound sites in 20 patients. Abbas Shafiee et al. [10] introduced a MEW-printed PCL mesh to mimic the anisotropic behavior of native skin tissue, that can be combined with attached and proliferated human gingival mesenchymal stem cells (hGMSCs). The use of cellular meshes resulted in wound healing with reduced scar formation compared to acellular ones, as tested in a rat model. In another study employing MEW, PCL ink was blended with milk protein to increase the biological functionality of the scaffold. Scaffolds in the shape of squares were printed, and human keratinocytes (HaCaTs) and normal human dermal fibroblast cells were seeded in the structure [17]. The scaffolds were proposed for use in the treatment of dermal wounds. To improve healing, biological molecules like growth factors can be added to the scaffolds [3], or nanoparticles (e.g. silver) as antimicrobial agents [18,19]. However, the fabrication of MEW-printed scaffolds loaded with biological molecules and nanoparticles for dermal fibroblast delivery has not been reported yet. We envision that such an approach would further increase the scaffolds' healing potential for different kinds of wounds.

Melt electrowriting can be used to fabricate promising scaffolds for wound healing. This method is an emerging additive manufacturing technique with high resolution and allows customized porosity adjusted to the specific application [20]. Only a limited number of thermoplastic polymers have been processed for MEW fabrication, including polycaprolactone (PCL) and its blends [[21], [22], [23], [24], [25]], poly-L-lactic acid (PLLA) [26], polypropylene (PP) [27], and polyvinylidene fluoride (PVDF) [28]. Poly-lactic acid (PLA) is a widely utilized material in tissue engineering due to its exceptional bio-absorbability, unmatched biocompatibility, and significant hydrolytic degradability [[29], [30], [31]]. However, until now, there have been only two research papers reporting on the MEW printing of PLLA scaffolds. Jie Meng et al. [26] printed 3D micro-fibrous PLLA scaffolds using MEW, with 200 μm pore size and 40 μm filament diameter, which was proposed for applications in bone tissue engineering. In a follow-up study [30], the authors MEW-printed PLLA, and additionally cast gelatine and bioglass inside the scaffold to achieve mechanical reinforcement [32].

Here, we propose to use MEW of PLA for printing a wound dressing. We decided to employ MEW technology as it allows to generate high-resolution fibrous and porous structures using thermoplastics [33]. Typically, for MEW the material needs to be melted in the cartridge for over an hour before printing. As a result, MEW-printing of PLA which is thermally sensitive, may be challanging. Yet, PLA has many excellent properties, such as good compatibility with human tissues and high durability [34] which motivates us to explore its potential for fabrication with MEW for wound dressing applications. To enhance PLA processability, thermal stability, and biocompatibility, nanodiamonds were incorporated by mixing them into the PLA solution prior to printing. After evaporating the solvent, the PLA/ND composite showed improved thermal properties compared to pure PLA, in accordance to the literature [35,36] revealing better printing performance and overall material stability in MEW applications.

Using optimized PLA/ND material, hierarchical scaffolds mimicking the real skin tissue were printed, with pore sizes from a minimum of 100 μm to a maximum of 300 μm and filament size of 17.12 ± 0.5 μm. To further improve the biological properties of the MEW-printed PLA/ND scaffolds, and achieve antibacterial activity, QβC was coated on the scaffolds. Chitin was chosen as it has been demonstrated before to accelerate wound repair and enhance cellular functions, such as the proliferation of human skin fibroblasts and keratinocytes [[37], [38], [39]]. Additionally, β-chitin has antimicrobial activities via positive charges. Indeed, after coating QβC on the scaffolds, the antibacterial properties of the scaffolds were confirmed and fast fibroblast proliferation was observed. All in all, the proposed cell-laden patches are promising for wound dressing applications.

## 2 Materials and methods

### 2.1 Preparation of PLA/ND material for printing

The PLA/ND material for printing was prepared as follows. Detonation nanodiamond powder (G01 grade NDs, Plasmachem GmbH, Germany) with a diameter of 4 nm was used. The preparation of ND suspension was carried out following the protocol introduced by Hees et al. [40] and Wehling et al. [41] In short, 1 g of the nanodiamond powder was annealed for 5 h at 450 °C in air to produce negatively charged NDs by the oxidation of the surface. The pretreated powder was dispersed in deionized water and ultrasonicated at 240 W for 1 h to obtain a ND dispersion at a concentration of 50 mg/mL. The dispersion was centrifuged at 4500 rpm for 20 min to remove large aggregates. 1.9 g PLA pellets (PLA, Purasorb PL 32, Corbion) were dissolved in 30 mL of 1,1,1,3,3,3-hexafluoro-2-propanol (HFP, Bioslove). 2 mL of 50 mg/mL nanodiamond dispersion were ultrasonicated for 1 h before mixing with PLA solution to get PLA/ND mixtures. The mixture was heated to 50 °C and stirred vigorously at 1000 rpm for 30 min to obtain a homogeneous solution.

Such mixtures were cast onto a glass dish and we evaporated HFP slowly at 60 °C for 1 h. To remove traces of HFP, the film was left in the fume hood for 2 days. The final ND concentration was 5 ± 0.4 wt.% (measured by scale inside of a TGA machine). Pristine PLA without nanodiamonds was used as a control printable material.

Prior to printing, the films were cut into small pieces and loaded into the cartridge. Air was removed from the cartridge by pumping in N_2_ at 2 bar for 5 min to minimize PLA hydrolysis [42]. Afterwards, the material was heated up to 220 °C and printed.

### 2.2 Physical properties of the PLA/ND composite material

#### 2.2.1 Measurements of mechanical properties

To assess the impact of nanodiamonds on the mechanical properties of the PLA/ND composite, Young's moduli, ultimate tensile strength, and elongation of pure PLA and PLA/ND films were analyzed using an Instron 5565 1 kN Series IX tensile tester with a 0.1 kN load cell. Samples with dimensions of 20 mm × 10 mm x 0.25 mm were prepared, and 3 samples for each experimental group were measured with the testing gauge at 5 mm and elongation speed at 10 mm/min.

#### 2.2.2 Thermal stability

To assess the usability of the composite material for MEW, and define the best temperature for material printing, thermal stability was analyzed. Melting points of pure PLA and PLA/ND films at 0, 0.5, 1, 2, and 3 h of preheating time were measured using a differential scanning calorimeter (DSC, PerkinElmer DSC-7). Specimens of 4–6 mg were precisely weighed by Quintix® Semi-Micro Balances and sealed in aluminum pans for the measurement. Then the samples were heated up from room temperature to 260 °C with a scanning speed at 20 °C/min under N_2_ flow of 15 mL/min followed by cooling to 100 °C and reheating to 260 °C.

The decomposition temperatures of PLA and ND-modified materials preheated for 0, 0.5, 1, 2, and 3 h were evaluated by using a thermogravimeter (TGA, Perkin-Elmer, TGA-7). 3–6 mg were weighed using the same scale, followed by scanning the samples from room temperature to 700 °C under nitrogen flow at 10 °C/min.

#### 2.2.3 Rheological property

Melt rheology at 220 °C under dynamical shear of the preheated polymer pellets (Ø 8 mm × 2 mm) was investigated with a rheometer (AR1000 N, TA) to compare rheological properties under different melting times. ^37^ A parallel plate (Ø = 8 mm, gap = 4 mm) geometry was selected for the dynamic frequency sweeps under a controlled strain of 5 %. This strain value was initially confirmed to be within the linear viscoelastic region for all the samples. The Environmental Test Chamber (ETC) was used and continuously purged with nitrogen, to analyze the material in conditions miming the MEW process. The angular frequencies were swept from 100 to 0.1 rad s^−1^.

#### 2.2.4 Molecular weight

Exclusion chromatography (SEC HPLC) measurements were conducted to assess the variation in molecular weight before and after heating, aiming to detect thermal degradation at different time points. The molecular weight of preheated PLA and PLA/ND samples in the cartridge was determined using a Hewlett-Packard instrument (HPLC series 1100) equipped with UV light and refractive index detectors. The separation was carried out by utilizing two PLGel 5 μm MIXED-C, 300 mm columns from Agilent Technologies at 35 °C. HPLC-grade chloroform was used as the eluent with a flow rate of 0.5 mL/min. Data acquisition and calculations were performed using Viscotek OmniSec software version 5.0. Molecular weights were determined based on a conventional calibration carried out with polystyrene standards. The samples were filtered over a 0.2 μm PTFE filter before injection.

### 2.3 Printing of pure PLA and PLA/ND materials

#### 2.3.1 Modification of the melt electrowritting device

A MEW device (Spraybase® A-1204-0001-01D, Ireland) was used, equipped with an additional hotplate fixed on the original collector in order to enhance the printing. A conductivity-isolated film and an earth wire were applied to connect the new collector to the ground. The heating temperature was set to 100 °C and the final temperature on the collector was around 80 °C.

#### 2.3.2 Scaffold fabrication

The PLA/ND films were melted at 220 °C in the stainless-steel syringe cartridge for 10 min, under a nitrogen environment. Then meshes with a strand spacing of 300 μm were printed using the setup described above. A final 6-layer structure designed by a SEL Program Generator (Dispense) (IAI Corporation, the USA) with 100–300 μm inter-fiber distance was printed. Pristine PLA without nanodiamonds was used as a control material. To print PLA and PLA/ND, a low voltage of 2.2–3.5 kV and pressure of 0.04–0.05 bar were used and adjusted accordingly to obtain the same fiber diameters for both materials (ca.17.12 ± 0.5 μm). The printing distance between the nozzle and the collector was kept at 2.5 mm and the collecting velocity was 150 mm/min.

### 2.4 Surface modification using quaternized β-chitin (QβC)

#### 2.4.1 Synthesis and characterization of quaternized β-chitin (QβC)

The QβC was synthesized according to previous studies [43]. β-Chitin was extracted from squid pens that were purchased at the market. The raw β-chitin was purified with 1 M aqueous NaOH overnight for deproteination and then with 1 M aqueous HCl overnight for demineralization at room temperature. The mixture was washed with deionized water between each step and the purification procedures were repeated twice. The purified β-chitin was lyophilized and ground into powder. To make QβC, 20 g KOH and 4 g urea were dissolved in 75 mL distilled water and precooled to −20 °C. Then, 1 g purified β-chitin was added immediately to the solution, followed by vigorous stirring for approximately 30 min to obtain a transparent solution. 16 g of 2, 3-epoxypropyl trimethylammonium chloride (EPTMAC) (Sigma) were added dropwise into 100 g of β-chitin solution, the mixture was stirred vigorously at 0 °C for 24 h. After that, the mixture was neutralized with aqueous HCl, dialyzed (MWCO≈3500 Da) against distilled water for 7 days, and then freeze-dried. ^1^H NMR (Bruker Ascend 600 FT-NMR, USA) and FTIR (Bruker IFS88, USA) were utilized to determine the chemical structure of QβC, and the surface charge was determined via zeta potential measurements (Malvern Panalytical Ltd, UK).

#### 2.4.2 Coating of PLA/ND scaffolds with QβC

The PLA/ND scaffolds were soaked in 5 wt.% QβC aqueous solution for 15 min, then rinsed in Dulbecco's phosphate-buffered saline (PBS, Sigma) to remove unattached QβC, followed by drying at room temperature for 1 day. PLA scaffolds with no NDs were treated using the same procedure to obtain control samples.

#### 2.4.3 Physical characterization of the scaffolds

The weight of PLA and PLA/ND scaffolds before and after QβC-coating was measured by a scale (Mettler Toledo 5-digit, the USA) with high accuracy. The release of the QβC on PLA and PLA/ND scaffolds was determined by energy-dispersive X-ray analysis, the detailed procedure is presented in SI.

Wettability of PLA, QβC coated PLA (further denoted as C/PLA), PLA/ND, PLA/ND coated with QβC (further denoted as C/PLA/ND) scaffolds were analyzed using a DataPhysics OCA30 (USA) system. 5 μL droplets of pure water (Milli-Q) were placed on the sample using an automated syringe, and images were taken. 3 measurements were performed on each sample and the average value was reported.

Morphological SEM images were captured using a field emission scanning electron microscope (FEI Nova NanoSEM 650 scanning electron microscope, the USA). The PLA specimens were coated with 15 nm gold using Ar plasma and investigated at 15 kV accelerating voltage under high vacuum conditions. The fiber diameters were measured from the SEM images using the Image J software (NIH, USA). Cross-section and high-magnification SEM images were taken using FEI Quanta 400 FEG at 3 kV and 10 kV accelerating voltages under high and low vacuum conditions.

Degradation experiments were performed to assess the stability of the PLA-based scaffolds. After the scaffolds were sterilized with 70 % ethanol, 500 μL cell medium (DMEM medium with 10 % FBS, and 1 % penicillin-streptomycin) was added to each well of a 24-well plate containing the PLA-based scaffolds. The surface morphology of the scaffolds after 3 months (medium was changed every 3 days) of incubation was imaged by SEM as mentioned before.

For transmission electron microscopy (TEM) the scaffolds were placed between two 400 mesh copper grids and visualized by a JEOL (Tokyo, Japan) JEM-2100 LaB_6_ transmission electron microscope at 200 kV accelerating voltage. Bright-field TEM images were acquired using a Gatan (Pleasanton, OR, USA) Orius SC1000 CCD camera.

Electrostatic surface potential (ESP) distribution was measured using a Kelvin probe force microscope (KPFM) on a Bruker Multimode 8 AFM system under ambient conditions [44]. Briefly, a Pt-coated silicon tip (cantilever resonance frequency ∼75 kHz; spring constant 2.8 N/m) was applied in lift mode with a lift height of 50 nm. PLA-based scaffolds were attached to a steel substrate via silver paste. The surface potential of the AFM tip was calculated by measuring a standard Au sample with a work function of 5.2 eV. The corresponding AFM images of PLA-based scaffolds are provided in Fig. SI 1.

### 2.5 Antibacterial evaluation of the PLA-based scaffolds

Before bacteria seeding, printed samples were sterilized using UV irradiation for 30 min. Then, the samples were washed 3 times using PBS. The antibacterial activities of the scaffolds against both *Staphylococcus aureus* and *Escherichia coli* were assessed using the proliferation assay [5] and a live/dead kit [45]. For the proliferation assay, the scaffolds (2 × 2 cm^2^) were placed in 6-well plates and co-incubated with Lysogeny broth (LB) medium for up to 24 h. The initial bacterial concentration was 1 × 10^7^ CFU/mL for both *S. aureus* and *E. coli*. At each time interval (0, 1, 2, 3, 4, 5, 6, 7, and 24 h), 0.1 mL bacterial LB medium from each group was extracted and its OD value at a wavelength of 600 nm was measured using a microplate reader (Thermo scientific Fluoroskan, Netherlands). LB medium without bacteria and LB medium with bacteria in well plates were taken as control groups. Three quadruplicate experiments were performed for each group. After 7 h, 1 mL of the bacterial suspensions was sent to centrifugation and washed twice with PBS. Then, a 20 μL mixture of SYTO 9 and propidium iodide (Sigma) in PBS (1:1 by volume) was added. Then we resuspended the bacteria, followed by incubation for 20 min at 37 °C in the dark. After washing three times in PBS, *S. aureus* and *E. coli* were resuspended in 1 mL PBS and transferred into confocal dishes. The stained specimens were imaged using a confocal microscope (Zeiss LSM 710, Germany).

### 2.6 Cell behavior on the PLA-based scaffolds

Normal Adult Human Dermal Fibroblast cells (NHDF-Ad cells, Lonza, The Netherlands) were used to test the biocompatibility of the scaffolds. The samples were sterilized using UV irradiation for 30 min and washed 3 times with PBS before cell seeding. For the preparation of cell cultures, sterile scaffolds with dimensions of 12 mm × 12 mm were placed into 24-well plates, and glass rings (sterilized with 70 % ethanol for 15 min and washed 3 times with PBS, Ø 12 mm × 2 mm, made by a workshop in the University of Groningen) were placed on top of the scaffold to prevent them from floating on the surface of the cell medium.

#### 2.6.1 Cell viability, cell numbers, metabolic activity and cell adhesion

The evaluation of cell viability was performed on the four scaffold types (PLA, C/PLA, PLA/ND, C/PLA/ND) using the following method. 1 mL of cell suspension at the density of 2.5 x 10^5^ cells/mL was seeded on a 15 mm × 15 mm scaffold and cultured in DMEM medium containing 10 % FBS and 1 % penicillin-streptomycin. After allowing the cells to adhere for 1 day, the cell-laden scaffolds were transferred into a new 24-well plate (to exclude from the analysis the cells adhering to the bottom of the original plates). The cells were cultured for up to 14 days in a 37 °C incubator under a 5 % CO_2_ atmosphere.

On days 1, 3, 7, and 14, cell viability was measured with the Fluorescein Diacetate/Propidium Iodide assay (FDA/PI, Sigma). In short, after aspiration of the cell culture medium, samples were washed 3 times with PBS. Cells in confocal petri dishes treated with 96 % ethanol and without any treatment were used as controls (Fig. SI 2a, b). 200 μL PBS solution containing 6 μg/mL FDA and 20 μg/mL PI were added to each sample and incubated for 15 min at room temperature. The staining reagent was removed and washed 3 times using PBS. The stained specimens were visualized and imaged using a confocal microscope (Zeiss LSM 710, Germany). Viability (%) = (number of live cells/total number of cells) x 100. Here, live cells are stained green, and dead cells are red. 10 images were used to do the calculation for each group.

Cell adhesion at day 1 on these scaffolds was collected as follows. 4′,6-diamidino-2-phenylindole (DAPI, Sigma) was applied to stain cell nuclei to do the counting. After washing with PBS, the samples were fixed with 4 % paraformaldehyde (PFA, sigma) for 10 min. Then samples were rinsed 3 times with PBS and permeabilized using 0.25 % triton X-100 (Thermo Fisher) for 10 min. Finally, 200 μL DAPI (5 μg/mL) was added to each well for 10 min, and cells were washed with PBS 3 times (5 min each wash). The cells and scaffolds were imaged with the same confocal microscope that was mentioned before. Nuclei stained in blue were counted using ImageJ. 10 images of each group were counted to gain average values.

The metabolic activity of the cells on scaffolds on days 1, 3, and 7 was tested, and cells seeded in Petri dishes served as control groups. In short, 20 μL of AlamarBlue was added to each sample into 180 μL of fresh culture medium (resulting in a total volume of 200 μl). Medium with 10 % AlamarBlue, with no contact with cells, was used as the negative control. Medium with 10 % AlamarBlue after 20 min of autoclaving was used as the positive control (full reduction). The solutions from samples and controls were extracted after 3-h incubation and transferred into 96-well plates. Relative fluorescence units (RFU) were measured by a microplate Fluorometer (Thermo scientific Fluoroskan, Netherlands) using 530 nm excitation and 590 nm emission wavelengths. AlamarBlue reduction in % (%ABr) was calculated by the following equation:

$$\%ABr=\frac{RFU_{samples}-RFU_{N}}{RFU_{P}-RFU_{N}}$$

Herein, RFU_samples_ means fluorescent data from samples, RFU_N_ and RFU_P_ refer to negative and positive controls, respectively [46].

##### 2.6.1.1 Immunostaining of cell attachment

The impact of 5 wt.% nanodiamond addition and subsequent scaffold's coating with QβC on cell attachment was tested on days 1, 3, and 7. After washing with PBS, the cells on the scaffolds were fixed with 4 % PFA for 10 min. Then, the cells were rinsed with PBS thrice and treated with 0.25 % triton X-100 for 10 min. To reduce nonspecific adhesion, the cells were blocked with bovine serum albumin (5 wt.% BSA, Sigma) for 30 min. Then, 100 μL of the primary antibody (10 μg/mL in PBS, Sigma) was used for immunostaining the cellular adhesion proteins (vinculin) by incubating the cells in a 4 °C refrigerator overnight. The remaining antibody solution was washed with PBS three times (5 min each time). 100 μL of the secondary antibody (5 μg/mL in PBS, Sigma) were added to each sample. Actin microfilaments were stained by using Phalloidin-iFluor 488 Reagent (Abcam) for 30 min. After washing three times (5 min each time, DAPI was applied to stain the cell nuclei.

#### 2.6.2 Immunofluorescent staining on cell contractility and extracellular matrix (ECM) remodeling

For the envisioned wound healing application, evaluation of cell contractility and extracellular matrix (ECM) remodeling on the scaffolds was necessary. Immunofluorescent staining was employed to investigate the expression of alpha-smooth muscle actin (α-SMA), which is known to stimulate fibroblast-mediated collagen contraction [47]. Additionally, the formation of collagen I, a crucial component in the skin remodeling process, was also stained for comparison. In short, after blocking, the fixed and permeabilized cells cultured on the scaffolds (day 3, 7, and 14) were incubated overnight in relevant primary antibodies at recommended concentration (Anti-alpha smooth muscle Actin antibody from Abcam, Collagen I polyclonal antibody from Thermo Scientific) at 4 °C. Then, we washed the samples and labeled them with appropriate secondary antibodies (Donkey-anti-mouse Red-X, and donkey-anti-rabbit from Sigma and, Abcam respectively) for 45 min using the suggested concentrations. To visualize the cells, we counterstained with phalloidin-TRITC (Sigma) and DAPI. Fluorescent images were captured using Zeiss 710.

## 3 Results and discussions

### 3.1 Physical properties of the pure PLA and PLA/ND composite

PLA is known to be a thermo-degradable polymer. Therefore, processing at elevated temperatures can have a high impact on physical properties including melting and decomposition as well as molecular weight and viscosity changes. For MEW printing, the complex viscosity (η*) of melts can determine the hydrodynamics of the Taylor cone which is the premise of steady printing. To obtain the physical properties of PLA and PLA/ND melts, the bulk materials (0.5 g) were added to the cartridge, subjected to different heating times at 220 °C, followed by physical material characterization to investigate variations of melting points, decomposition temperature, molecular weight, and complex viscosity.

**Fig. 1:**
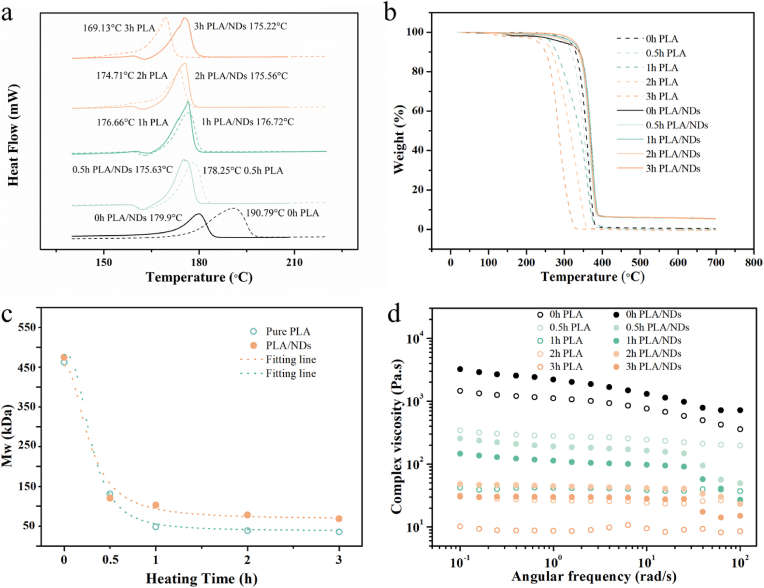
Physical properties of preheated PLA and PLA/ND composite for 0, 0.5, 1, 2, and 3 h. a) Heating curve of DSC. b) Weight loss and decomposition temperature shown by TGA. c) Variation of the molecular weight. d) Curve of complex viscosity dependence on angular frequency.

Fig. 1a shows the melting points of PLA and PLA/ND materials at various preheated times. The unheated PLA exhibited a melting peak at 190.79 °C. However, after heating for 0.5 h, the melting peak decreased to 178.25 °C. Subsequently, there was a slight decrease in the melting points from 0.5 h to 2 h of heating, from 178.25 °C to 174.71 °C. In turn, for PLA/ND the change in melting point was less pronounced. We observed a slight decrease from 179.90 °C to 175.22 °C in the melting point after 3 h of heating. This illustrates that with the incorporation of 5 wt.% NDs, the temperature required for material melting decreased by 10.89 °C, and the material exhibited increased stability during the heating process. Further, we observed that unprocessed PLA showed a 10 % weight loss at 340 °C (Fig. 1b). For the sample after different preheating times at 220 °C, for the 1-h heating group, 10 % weight loss was observed around 290 °C, and after the 3-h preheating, the sample underwent 10 % weight loss at 250 °C. This indicates the degradation of PLA samples under elevated temperatures. Compared to pure PLA samples, the PLA/ND composite showed increased stability under the same processing condition. The weight loss graphs show smaller differences between samples subjected to prolonged heating. All the samples lost 10 % of weight at 350 °C. This result is consistent with DSC data, indicating increased material thermal stability after ND addition. Finally, the SEC analysis showed that the molecular weight (Mw) of the PLA/ND composite and pure PLA decreased from 470 kDa to 120 kDa (Fig. 1c), within the initial 0.5 h of heating. Between 0.5 and 3 h, the PLA/ND composite displayed a minimal change in Mw. However, the Mw of PLA dropped by around 50 % during the subsequent 0.5 h (from 120 kDa to 50 kDa), followed by slight changes from 1 h to 3 h. Therefore, the addition of NDs is beneficial for MEW printing.

The changes in Mw were expected to influence observed complex viscosity. Indeed, the effect was visible based on the complex viscosity analysis (Fig. 1d). The initial viscosity of the PLA/ND (3.75 kPa s) composite was higher by 2 kPa s than pure PLA (1.75 kPa s), which

we assign to the interconnection between nanodiamonds and polymer chains. After different preheating times, the viscosity of the PLA/ND groups remained higher than that of the PLA samples, except for the group preheated at 0.5 h. Additionally, a notable drop was observed between 0.5-h and 1-h of heating for both groups (PLA group is from 380 Pa s to 45 Pa s, PLA/ND group is from 300 Pa s to 150 Pa s). Subsequently, the viscosity drops for the PLA/ND samples decreased within 3 h of heating, while for the PLA samples, the viscosity still dropped significantly from 2 h to 3 h of heating. NDs can act as a crystallization nucleus, enhancing the crystallization of PLA. The viscosity also increases with the inclusion of NDs, impeding crystallization. These opposing effects are presumed to result in the increase in the melting point discussed before [48]. In a previous study, Jie Meng et al. [26] computationally investigated the variation in molecular weight (Mw) of pure PLLA after preheating at 190 °C for 0–4 h. The simulations revealed that there was a significant drop (10.8 %) in the Mw after heating for 1 h, followed by a gentler decline in subsequent measurements, which is in line with our results. They simulated a viscosity reduction of 10.80 % after 2 h of heating, and this reduction increased to 15.26 % after 4 h of heating, but they did not provide measured viscosity data. In turn, for our system, we already observed a significant decrease in Mw after 0.50 h of heating in the cartridge. The results indicate that heating has an influence on the material's stability and viscosity, influencing the printing process of PLA and PLA/ND materials, even at short times. The effect is more pronounced for PLA than for PLA/ND and for times longer than 2 h. As MEW requires heating for prolonged times, based on the material analysis, the printing time should be shorter than 2 h.

Tensile moduli were used to compare the mechanical properties of PLA and PLA/ND materials. As the MEW printed scaffolds were very fragile, films were used for analysis. The results shown in Fig. SI 3 indicate a slight increase in the PLA/ND group on Young's modulus, ultimate tensile strength, and extension. This non-significant increase is in agreement with previous studies [35]. The improvements in the mechanical properties of the PLA were attributed to the homogeneous dispersion of NDs, the combination of NDs with PLA chains, and the strong adhesion between PLA and NDs.

Based on these findings, it is evident that the thermal stability was significantly modified with the addition of 5 wt% NDs in PLA with improved mechanical properties of PLA materials. This can be attributed to the interconnection between well-dispersed ND particles and polymer chains, as reported before [49]. Based on the results, for the MEW process, the materials were preheated in the cartridge for 30 min and subsequently printed for up to 1 h, after which the material was refreshed.

### 3.2 Physicochemical characterization of QβC and scaffolds

#### 3.2.1 Characterization of QβC

**Fig. 2:**
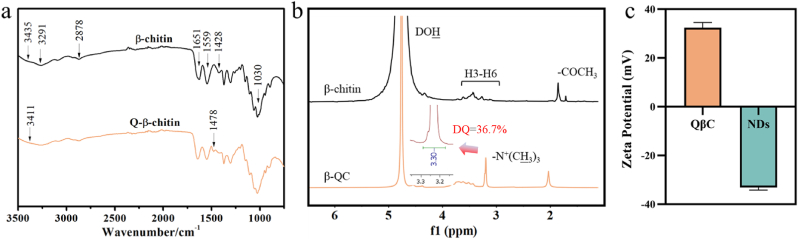
Physicochemical characterization of materials. a) FTIR spectra and b) ^1^H NMR spectra of QβC and untreated β-chitin, the degree of quaternization of QβC is 36.7 %. c) Zeta potential of QβC and NDs.

To verify the chemical structure of QβC, FTIR and ^1^H NMR characterizations were performed (see Fig. 2a and b). The FTIR spectrum of the purified squid pens contained the characteristic absorption peaks of β-chitin at 3435 cm^−1^ for OH stretching, 3291 cm^−1^ for NH stretching, 1651 cm^−1^ for the amide I band (υ(C=O)) and 1559 cm^−1^ for the amide II band (δ(N–H)). After quaternization, amide I and amide II bands were unaffected, whereas the OH stretching absorption peak shifted from 3435 to 3411 cm^−1^, and the new, characteristic absorption peak of the C–H bending of –N^+^(CH_3_)_3_, appeared at 1478 cm^−1^, showing the existence of quaternary ammonium groups in QβC. This functionalization was also consistent with the ^1^H spectra of QβC obtained by ^1^H NMR analysis. QβC and β-chitin were analyzed in NaOD (Sigma) solution. The hydroxyl protons of the N-acetyl-glucosamine residue contributed to the DOH signal produced by deuteration with NaOH/D_2_O. The signals of protons in the glucopyranose ring ranged from 2.23 to 3.80 ppm, and the protons of the –N^+^(CH_3_)_3_ group appeared in the new peak at 3.18 ppm, confirming the formation of quaternized β-chitin. The methyl protons of the acetamido groups appeared at 1.85 and 1.72 ppm, while the protons from QβC located at 2.02 ppm. That might be caused by esterification between chitin, EPTMAC, and deacetylation during the quaternization process. The degree of quaternization (DQ) of QβC at 36.7 % was calculated following the protocol reported before [50]. This DQ is similar to the 33 % reported in the literature [43]. The analysis of FTIR and NMR spectra proved that the EPTMAC was successfully grafted to the β-chitin structure, which was corroborated The zeta potential of QβC was 36.5 ± 0.5 mV, and the zeta potential of the ND was −34.1 ± 1.0 mV (Fig. 2c). Thus, we envisioned that the coating of QβC on PLA/ND scaffolds could be stabilized by electrostatic attraction between QβC and NDs.

#### 3.2.2 Characterization of scaffolds

**Fig. 3:**
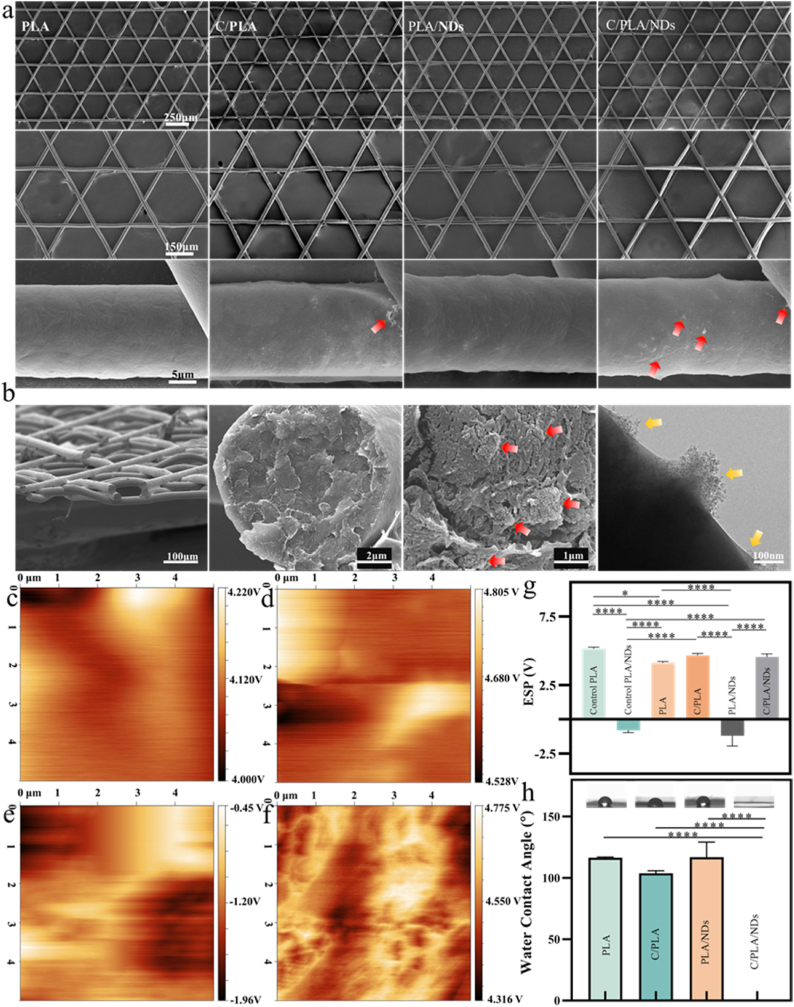
a) Macroscopic views and corresponding SEM images of PLA, C/PLA, PLA/ND, and C/PLA/ND scaffolds, images of low magnification show the uniformity of the printed scaffolds, the images at high magnification show the surface roughness of single fiber. b) Cross-section SEM and TEM images of C/PLA/ND scaffolds. These images indicate the decent distribution of NDs inside and on the surface of PLA fibers. c-g) KPFM measurement of surface potential distribution on PLA-based scaffolds: c) PLA, d) QβC-coated PLA, e) PLA/ND, f) QβC-coated PLA/ND. g) ESP of control samples PLA and PLA/ND melts and PLA, QβC-coated PLA, PLA/ND, QβC-coated PLA/ND scaffolds. h) Wettability of scaffolds. The result shows a sharp increase in hydrophilicity of the C/PLA/ND scaffold compared to other groups.

Scanning electron microscopy (SEM) images show the morphology of the 6-layered scaffolds printed with MEW. The uniform scaffolds with a fiber diameter of 17.12 ± 0.5 μm were produced with PLA and PLA/ND (Fig. 3a) and, subsequently, coated with QβC. The printability of PLA in comparison to PLA/ND was hindered by the incorporation of NDs at high concentrations (5 wt.%). Images at high magnification showed the fiber surfaces before and after coating (Fig. 3a). The PLA/ND fibers exhibited more QβC than pure PLA fibers (the QβC particles are highlighted by red arrows in Fig. 3a). Fig. 3b depicts a cross-sectional view of the QβC-coated PLA/ND scaffolds. Additionally, high-magnification SEM and TEM images showcase the ND agglomerates within the fibers (red arrows in Fig. 3b) and on the fiber surface (yellow arrows in Fig. 3b), which indicates increased absorbance of positively charged QβC.

The degradation measurements were performed to test the stability of the PLA-based scaffolds. After 3 months of incubation in cell medium, the SEM images of scaffold structure showed no difference between the initial samples and the medium-treated samples (Fig. 3a and Fig. SI 4). This indicates that the scaffolds are stable for several months, which is in agreement with a previous study showing the slow degradation of PLA [50]. The PLA-based scaffolds have a high potential to be applied in chronic wounds including pressure ulcers, diabetic foot ulcers, and venous leg ulcers [51], which require longer healing time and, therefore, longer stability of the scaffolds.

Surface potential can affect the biological properties of the scaffolds, especially cell adhesion and antibacterial properties. As cell and bacteria membranes carry negative charges, they tend to adhere to surfaces with positive charges. High positive charges are utilized to eliminate bacteria [51,52]. To determine the final surface potential of the prepared scaffolds, KPFM measurements were conducted. As shown in Fig. SI 5, Fig. 3c and 3g, PLA melts and PLA scaffolds carry positive charges, with a surface potential of 5.17 V and 4.11 V, respectively. PLA can be positively charged when there are no negatively charged molecules or ions in its vicinity to counterbalance the positive charges [53,54]. Due to the incorporation of negatively charged nanodiamonds in PLA, the PLA/ND melt initially exhibited negative charges (Fig. 3e–g). According to a previous study, the inflight fibers coming from the nozzle in the air carry positive charges [55]. However, when the layer number is no more than 30 (here, we have 6 layers), the collected filaments are very likely to be more negative due to the rapid dissipation of positive charges [54,55]. This explains why the extruded MEW PLA/ND scaffolds (−1.2 V) have a higher negative charge than PLA/ND melt (−0.76 V) in Fig. 3g. After coating PLA/ND scaffolds with QβC, the surface potential shifted back to the positive range, reaching a value similar to that of coated and uncoated PLA scaffolds (Fig. 3c–g). According to the measurement on weight shift, the PLA/ND scaffolds achieve a higher QβC absorption (0.26 mg QβC/1.35 mg scaffold, 19.26 wt.%) to exhibit a comparable excess of positive charges compared to that of the PLA group (0.15 mg QβC/1.33 mg scaffold, 11.28 wt.%). QβC quantity is more obvious in the C/PLA/ND group, which is in good agreement with the SEM images.

The water contact angle was measured to indicate the scaffold's surface wettability (Fig. 3h), which has an impact on protein absorption, cell adhesion, and subsequent cell spreading and proliferation [56]. With the QβC coating, the water contact angle of the PLA/ND scaffolds exhibited a significant decrease, from 116.5° (without coating) to 0°, which indicated that the scaffolds changed from high hydrophobicity to complete hydrophilicity. The enhanced hydrophilicity can be most probably explained by the abundant hydroxyl and amino groups on QβC's backbone [57], and the increase in hydrophilicity may result from the higher QβC absorption. It was suggested before, that on surfaces with proper wettability, the adhesive proteins can effectively replace other proteins, leading toincreased cell attachment [58]. This indicates a potential benefit of QBC coatings for ] attachment and spreading.

### 3.3 Antibacterial evaluation

**Fig. 4:**
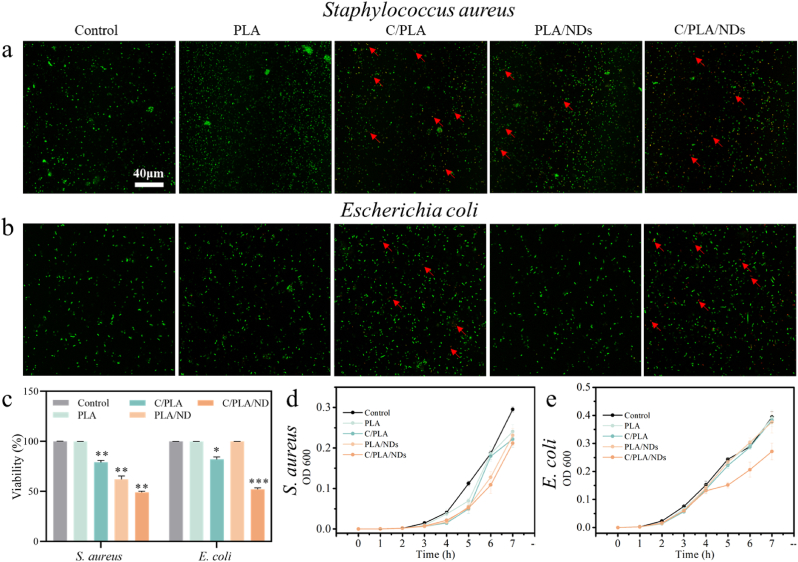
a) and b) Live/dead staining of *S. aureus* and *E. Coli* on these scaffolds after 7-h co-incubation. Dead bacteria shown as red dots were highlighted by red arrows. c) Viability of the two bacteria on PLA-based scaffolds after 7 h. d) and e) the proliferation at various times up to 7 h of co-culture of *S. aureus* and *E. coli* with scaffolds. Here * indicates p < 0.05, ** means p < 0.01, *** means p < 0.001 and **** means p < 0.0001. (For interpretation of the references to color/colour in this figure legend, the reader is referred to the Web version of this article.)

Wound infections and inflammation caused by bacteria would impede the natural healing process. Yet, traditional wound dressing patches have no antimicrobial properties to eliminate the microorganisms or stop their growth. To observe the antimicrobial properties of our scaffolds, the proliferation and viability of *Staphylococcus aureus* (*S. aureus*) and *Escherichia coli* (*E. coli*) on our printed scaffolds were measured. The results of microbial viability tests of *S. aureus*, and *E. coli* after incubation with scaffolds for 7 h are shown in Fig. 4a, b, and c. SYTO 9 stains live cells (green) which combines with intracellular esterase, while propidium iodide (PI) binds to the DNA of dead membrane-compromised cells (red dots). 50 % of both bacteria were killed on QβC-treated PLA/ND scaffolds, and 20 % on C/PLA scaffolds, whereas in the PLA group, the viability of bacteria was very high (almost 100 %) after 7 h (Fig. 4c). This revealed the antimicrobial properties of QβC-coated scaffolds. The viability of the two bacteria types on PLA/ND scaffolds is different, *S. aureus* is 60 %, however, 100 % for *E. coli*. The proliferation rates of both bacteria were the lowest on PLA/ND scaffolds with a QβC coating layer in the culture longer than 5 h. Based on the energy-dispersive X-ray spectroscopy (EDX) analysis of the samples (determined by the Nitrogen content) limited amount of QβC was left on PLA samples after 5 h (Fig. SI 4), explaining the decrease of inhibition of S. Aureus growth on C/PLA (Fig. 4d). Whereas for most of the timepoints more QβC was attached to PLA/ND than to pure PLA (Fig. SI 6) (and antimicrobial effect was more pronounced), indicating the NDs enhance the binding between the coating material with scaffolds. The coated PLA/ND scaffolds, containing both NDs and QβC, exhibited antibacterial effects against *S. aureus* and *E. coli* within 7 h (Fig. 4 a-e). Combined with the survival rate, the PLA/ND scaffolds also showed inhibition on the proliferation of *S. aureus* after 7-h co-incubation (Fig. 4a–d). We assign this effect to a little antibacterial property of NDs according to previous reports [4,41]. We anticipated that in our scaffolds, besides the partially oxidized NDs, the antibacterial activities resulted from the positive charges of QβC. The positively charged QβC interacts with the negatively charged bacteria wall and then damages the membrane permeability and kills bacteria [43,59,60].

Importantly, after 24-h coincubation (Fig. SI 7) the bacterial viability was nearly 100 %, indicating that the antibacterial effect is short-termed andlimited to an initial ca. 7 h. Although the antibacterial effect is mild, it could be enhanced in the future by increasing the quantity and stability of QβC on the scaffolds.

### 3.4 Cell behavior

#### 3.4.1 Cell viability, adhesion, metabolism and attachment

**Fig. 5:**
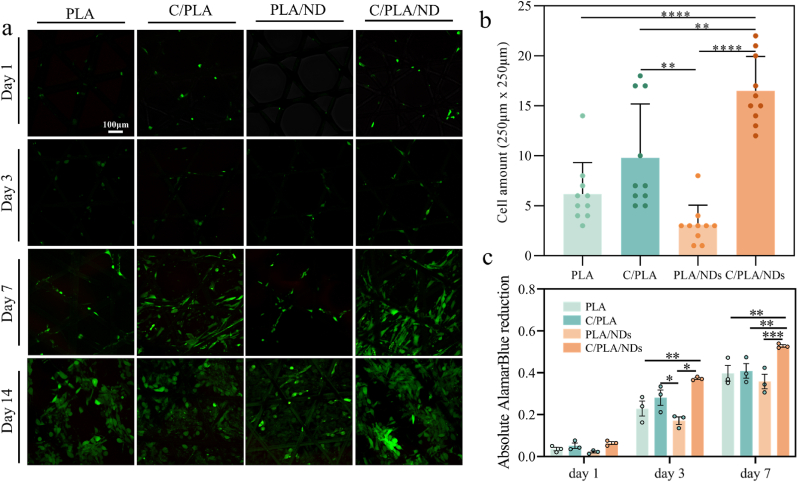
Cell performance on the printed scaffolds. a) Live and dead staining (green for live cells and red means dead cells) of NHDF-Ad cells on scaffolds after 1-day, 3-day, 7-day, and 14-day cultures to assess cell viability. b) Cell numbers on these scaffolds at day 1 to assess initial cell attachment. c) Cell metabolic activity on the scaffolds growing after day 1, day 3, and day 7 tested by AlamarBlue assay. (For interpretation of the references to color/colour in this figure legend, the reader is referred to the Web version of this article.)

In the next step, we assessed the cytocompatibility of the scaffolds with human dermal fibroblasts to evaluate their utility for wound-healing grafts. A live/dead assay (FDA/PI) was performed to measure the viability of NHDF cells on the four different scaffolds after 1, 3, 7, and 14 days of incubation (Fig. 5a). There was no red fluorescence detected on these scaffolds, indicating no cytotoxicity to the dermal fibroblasts caused by the addition of 5 wt.% NDs or QβC to PLA.

To quantify the cell attachment, cell numbers on the scaffolds were counted after labeling with DAPI and imaging with the confocal microscope. The results are presented in Fig. 5b. Apparently, on day 1, the QβC-coated PLA/ND scaffolds presented the highest cell attachment, which could be explained by the positive charges and high wettability of those scaffolds (Fig. 3g and h). On pure PLA and PLA/ND scaffolds, fewer cells were attached compared to QβC-coated PLA-based scaffolds. The non-coated PLA/ND scaffolds showed the lowest cell adhesion, which may result from the negative charges, leading to the repulsion of the negative charges in the cell membrane.

Metabolic activities of the cells on the scaffolds were further compared using AlamarBlue (see Fig. 5c). After a 3-day culture, the metabolism of the QβC groups exhibited a significant statistical difference (*p < 0.05) in comparison with non-treated PLA/ND samples. The coated PLA/ND scaffolds showed the highest cell activity after one week of incubation. Although the PLA/ND scaffolds had compromised cell attachment, the proliferation of the existing cells was comparable to other groups. We observed that QβC is beneficial for cell attachment and metabolism, a similar effect of chitin and chitosan was observed before by Howling et al. [39].

**Fig. 6:**
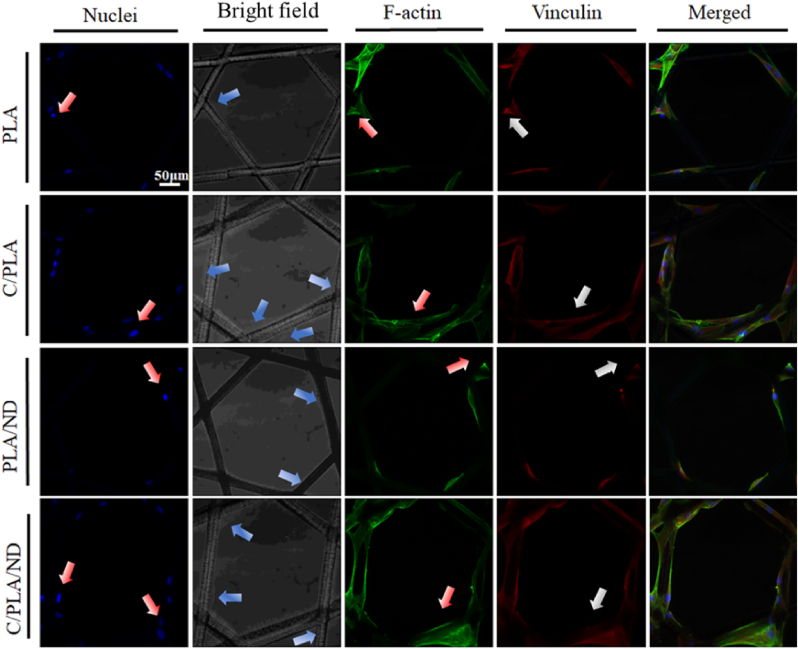
Cell attachment on the scaffolds at day 1. Cell nuclei (blue, marked with red arrows), F-actin (green, marked with red arrows), and focal adhesive protein-vinculin (red, marked with white arrows) were stained to show the cell adhesion on different scaffolds. Scale bar: 50 μm. Bright-field images of the scaffolds are presented with increased brightness (40 %) for better visibility. (For interpretation of the references to color/colour in this figure legend, the reader is referred to the Web version of this article.)

To gain further information on cell spreading, focal adhesions (FA) of the cells on scaffolds on day 1, 3, and 7 were compared by looking at the morphology of nuclei, F-actin, and vinculin, respectively (Fig. 6). The shapes of nuclei were round (red arrows), in some of the cells, the area of F-actin (red arrows) and vinculin (white arrows) were small and aligned without well-defined direction, indicating FA were immature, focal adhesions are structures that mechanically link F-actin fibers with the extracellular substrate through proteins such as vinculin [[61], [62], [63]]. The elongated FA shape indicates cell maturation and strong adhesion is formed between cells and scaffolds [64,65]. Cell nuclei were elliptical and cells were well spread on QβC-coated PLA-based scaffolds. The cells had elongated shapes with more spindle-shaped F-actin fibers and vinculin aligned on QβC-coated fibers when compared to the cells on pure PLA and PLA/ND scaffolds, suggesting maturation and strong adhesion of cells in contact with these scaffolds. Results from day 3 (Fig. SI 9), and 7 (Fig. SI 10) also suggest that the QβC coating improved cell focal adhesion area and size as well compared to cells on pure PLA and

PLA/ND. The cell nuclei are bigger and more elliptical than they are on pure PLA and PLA/ND scaffolds. A confluent layer was first formed on the coated PLA/ND scaffolds after one week. The results of ND addition on cell spreading were proved by several papers as well. Lubica et al. [66] reported enhanced adhesion, growth, and differentiation of bone cells on nanostructured

nanocrystalline diamond (NCD) films compared to control polystyrene culture dishes. Marie et al. [67] compared cell attachment and spreading area on pure silicon oxide surfaces and surfaces coated with oxidized nanocrystalline diamond (O-NCD) films, revealing improved cell adhesion and larger spreading area with O-NCD. As there is limited information on the effect of QβC on cell adhesion and spreading in previous studies, our findings provided valuable insights into cell behavior in the presence of QβC.

#### 3.4.2 Contractility and extracellular matrix (ECM) remodeling

In order to obtain more comprehensive information about the contraction and remodeling profile of the cellular scaffolds, we compared the expression of wound contraction protein (α-SMA) and extracellular matrix (ECM) remodeling protein (collagen I) through immunostaining from day 3 to day 14 (Fig. SI 11, 7 and SI 12). Representative results on day 7 are presented in

**Fig. 7:**
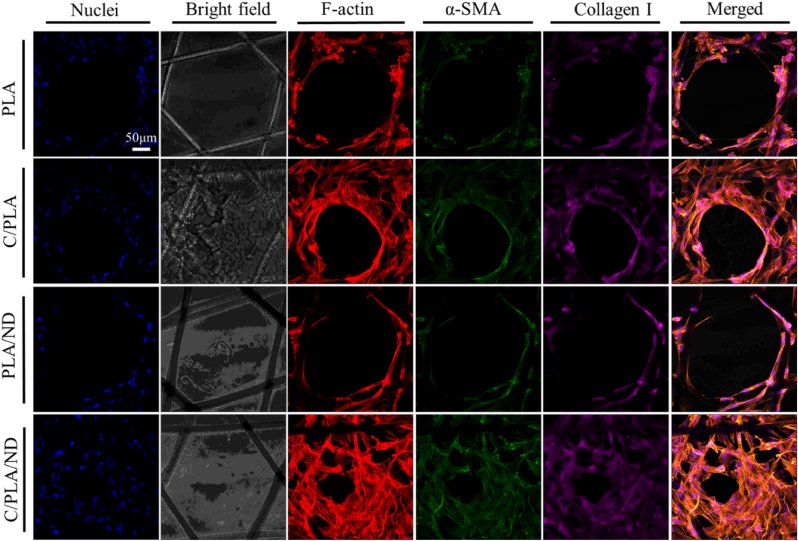
Expression of wound contraction protein (Alpha -SMA in green) and ECM remodeling protein (collagen I) on the different examined scaffolds at day 7. Cell nuclei were marked in blue, the gray images showed a bright field, and F-actin was labeled in red. Scale bar: 50 μm. Bright-field images of the scaffolds are presented with increased brightness (40 %) for better visibility. (For interpretation of the references to color/colour in this figure legend, the reader is referred to the Web version of this article.)

Fig. 7. As shown, cells on coated PLA and coated PLA/ND scaffolds expressed thicker α-SMA with larger spreading areas in regions that have denser cell numbers, suggesting higher fibroblast activation and differentiation [68]. α-SMA which is expressed in myofibroblasts, activated and differentiated fibroblasts [69] is beneficial to the contraction [70]. The balanced wound contraction facilitates tissue repair. Too little contraction causes non-healing wounds, whereas too much of it leads to scars. Our scaffolds were able to provide the right physical environment to adjust the contraction of the wounds. A more homogeneous and denser production and organization of collagen I was observed in the scaffolds with QβC and NDs, which can be assigned to the synergetic effect of these two bioactive cues. The decreased level of collagen I would contribute to the non-healing state and a reduction in tensile strength, which has been observed in chronic wounds [71]. The highest expression of ECM proteins is observed on C/PLA/ND scaffolds in Fig. 7. The expression is more prominent at the interface between cells and the medium, as indicated by the position of F-actin in all experimental groups. It revealed that the well-distributed and stable QβC on the fibers could induce faster ECM production which is needed for the closure of the wound. The results from day 3 (Fig. SI 11) suggest a similar tendency. However, after 2 weeks, there is no significant difference between these groups in collagen expression (Fig. SI 12). Summarizing, with the help of the scaffold structure and the addition of QβC and NDs, the fast formation of a neo-dermis layer could be obtained.

## 4 Conclusion

The majority of dressing materials reported before on wound healing were focused on modification of the materials and their function using biologically active molecules. Fewer studies combine the use of cells, bioactive molecules, and scaffolds [72,73]. We have successfully printed high-resolution PLA/ND scaffolds using MEW. After coating with QβC, the scaffolds showed increased hydrophilicity and short-term antimicrobial properties. Further, the scaffolds exhibited excellent biocompatibility, including improved cell metabolism, attachment, contraction, and remodeling of ECM. In conclusion, our result provides an interesting approach for producing wound dressing scaffolds using MEW that can deliver cells and biologically active molecules simultaneously, which renders them promising for the treatment of various wound sites.

## Funding

This work was supported by the 10.13039/501100004543China Scholarship Council (202006260009, 2020); and the 10.13039/501100003246Dutch Research Council (Veni grant no.VI.Veni.192.148, 2020).

## CRediT authorship contribution statement

**Xixi Wu:** Writing – review & editing, Writing – original draft, Visualization, Validation, Supervision, Software, Project administration, Methodology, Investigation, Formal analysis, Data curation, Conceptualization. **Wenjian Li:** Writing – review & editing, Methodology, Formal analysis. **Lara Herlah:** Investigation. **Marcus Koch:** Writing – review & editing, Investigation. **Hui Wang:** Investigation. **Romana Schirhagl:** Writing – review & editing, Supervision, Software, Resources, Project administration, Funding acquisition. **Małgorzata K. Włodarczyk-Biegun:** Writing – review & editing, Supervision, Project administration, Methodology, Funding acquisition.

## Declaration of competing interest

The authors declare that they have no known competing financial interests or personal relationships that could have appeared to influence the work reported in this paper.

## Data Availability

Data will be made available on request.

## References

[1] Eming S.A., Martin P., Tomic-Canic M. (2014). Wound repair and regeneration: mechanisms, signaling, and translation. Sci. Transl. Med..

[2] Shi C., Wang C., Liu H., Li Q., Li R., Zhang Y. (2020). Selection of appropriate wound dressing for various wounds. Front. Bioeng. Biotechnol..

[3] Alizadehgiashi M., Nemr C.R., Chekini M., Pinto Ramos D., Mittal N., Ahmed S.U. (2021). Multifunctional 3D-printed wound dressings. ACS Nano.

[4] Houshyar S., Kumar G.S., Rifai A., Tran N., Nayak R., Shanks R.A. (2019). Nanodiamond/poly-ε-caprolactone nanofibrous scaffold for wound management. Mater. Sci. Eng. C.

[5] Hu W., Wang Z., Zha Y., Gu X., You W., Xiao Y. (2020). High flexible and broad antibacterial nanodressing induces complete skin repair with angiogenic and follicle regeneration. Adv. Healthcare Mater..

[6] Desjardins-Park H.E., Foster D.S., Longaker M.T. (2018). Fibroblasts and wound healing: an update. Regen. Med..

[7] Wall I.B., Moseley R., Baird D.M., Kipling D., Giles P., Laffafian I. (2008). Fibroblast dysfunction is a key factor in the non-healing of chronic venous leg ulcers. J. Invest. Dermatol..

[8] Cha J., Kwak T., Butmarc J., Kim T.A., Yufit T., Carson P. (2008). Fibroblasts from non-healing human chronic wounds show decreased expression of βig-h3, a TGF-β inducible protein. J. Dermatol. Sci..

[9] Kirby G.T.S., Mills S.J., Cowin A.J., Smith L.E. (2015). Stem cells for cutaneous wound healing. BioMed Res. Int..

[10] Shafiee A., Cavalcanti A.S., Saidy N.T., Schneidereit D., Friedrich O., Ravichandran A. (2021). Convergence of 3D printed biomimetic wound dressings and adult stem cell therapy. Biomaterials.

[11] Zaulyanov L., Kirsner R.S. (2007). A review of a bi-layered living cell treatment (Apligraf) in the treatment of venous leg ulcers and diabetic foot ulcers. Clin. Interv. Aging.

[12] Gibbons G.W. (2015). Grafix ® , a cryopreserved placental membrane, for the treatment of chronic/stalled wounds. Adv. Wound Care.

[13] Landsman A., Rosines E., Houck A., Murchison A., Jones A., Qin X. (2016). Characterization of a cryopreserved split-thickness human skin allograft-TheraSkin. Adv. Skin Wound Care.

[14] Phillips T.J., Manzoor J., Rojas A., Isaacs C., Carson P., Sabolinski M. (2002). The longevity of a bilayered skin substitute after application to venous ulcers. Arch. Dermatol..

[15] Da Silva L.P., Reis R.L., Correlo V.M., Marques A.P. (2019). Hydrogel-based strategies to advance therapies for chronic skin wounds. Annu. Rev. Biomed. Eng..

[16] Yoshikawa T., Mitsuno H., Nonaka I., Sen Y., Kawanishi K., Inada Y. (2008). Wound therapy by marrow mesenchymal cell transplantation. Plast. Reconstr. Surg..

[17] Hewitt E., Mros S., McConnell M., Cabral J.D., Ali A. (2019). Melt-electrowriting with novel milk protein/PCL biomaterials for skin regeneration. Biomedical Materials (Bristol).

[18] Dizaj S.M., Lotfipour F., Barzegar-Jalali M., Zarrintan M.H., Adibkia K. (2014). Antimicrobial activity of the metals and metal oxide nanoparticles. Mater. Sci. Eng. C.

[19] Rai M., Yadav A., Gade A. (2009). Silver nanoparticles as a new generation of antimicrobials. Biotechnol. Adv..

[20] Kade J.C., Dalton P.D. (2021). Polymers for melt electrowriting. Adv. Healthcare Mater..

[21] Castilho M., Feyen D., Flandes-Iparraguirre M., Hochleitner G., Groll J., Doevendans P.A.F. (2017). Melt electrospinning writing of poly-hydroxymethylglycolide-co-ε-caprolactone-based scaffolds for cardiac tissue engineering. Adv. Healthcare Mater..

[22] Paxton N.C., Ren J., Ainsworth M.J., Solanki A.K., Jones J.R., Allenby M.C. (2019). Rheological characterization of biomaterials directs additive manufacturing of strontium-substituted bioactive glass/polycaprolactone microfibers. Macromol. Rapid Commun..

[23] Bai J., Wang H., Gao W., Liang F., Wang Z., Zhou Y. (2020). Melt electrohydrodynamic 3D printed poly (ε-caprolactone)/polyethylene glycol/roxithromycin scaffold as a potential anti-infective implant in bone repair. Int. J. Pharm..

[24] Hochleitner G., Kessler M., Schmitz M., Boccaccini A.R., Tebmar J., Groll J. (2017). Melt electrospinning writing of defined scaffolds using polylactide-poly(ethylene glycol) blends with 45S5 bioactive glass particles. Mater. Lett..

[25] Abdal-hay A., Abbasi N., Gwiazda M., Hamlet S., Ivanovski S. (2018). Novel polycaprolactone/hydroxyapatite nanocomposite fibrous scaffolds by direct melt-electrospinning writing. Eur. Polym. J..

[26] Meng J., Boschetto F., Yagi S., Marin E., Adachi T., Chen X. (2021). Design and manufacturing of 3D high-precision micro-fibrous poly (L-lactic acid) scaffold using melt electrowriting technique for bone tissue engineering. Mater. Des..

[27] Haigh J.N., Dargaville T.R., Dalton P.D. (2017). Additive manufacturing with polypropylene microfibers. Mater. Sci. Eng. C.

[28] Florczak S., Lorson T., Zheng T., Mrlik M., Hutmacher D.W., Higgins M.J. (2019). Melt electrowriting of electroactive poly(vinylidene difluoride) fibers. Polym. Int..

[29] Mondal S., Nguyen T.P., Pham V.H., Hoang G., Manivasagan P., Kim M.H. (2020). Hydroxyapatite nano bioceramics optimized 3D printed poly lactic acid scaffold for bone tissue engineering application. Ceram. Int..

[30] Shuai C., Yang W., Feng P., Peng S., Pan H. (2021). Accelerated degradation of HAP/PLLA bone scaffold by PGA blending facilitates bioactivity and osteoconductivity. Bioact. Mater..

[31] Feng P., Wu P., Gao C., Yang Y., Guo W., Yang W. (2018). A multimaterial scaffold with tunable properties: toward bone tissue repair. Adv. Sci..

[32] Meng J., Boschetto F., Yagi S., Marin E., Adachi T., Chen X. (2022). Melt-Electrowritten Poly(L-lactic acid)- and Bioglass-Reinforced biomimetic hydrogel for bone regeneration. Mater. Des..

[33] Reizabal A., Kangur T., Saiz P.G., Menke S., Moser C., Brugger J. (2023). MEWron: an open-source melt electrowriting platform. Addit. Manuf..

[34] Ranakoti L., Gangil B., Bhandari P., Singh T., Sharma S., Singh J. (2023). Promising role of polylactic acid as an ingenious biomaterial in scaffolds, drug delivery, tissue engineering, and medical implants: research developments, and prospective applications. Molecules.

[35] Zhao Y.Q., Lau K.T., Kim J.K., Xu C.L., Zhao D.D., Li H.L. (2010). Nanodiamond/poly (lactic acid) nanocomposites: effect of nanodiamond on structure and properties of poly (lactic acid). Compos. B Eng..

[36] Mochalin V.N., Gogotsi Y. (2015). Nanodiamond-polymer composites. Diam. Relat. Mater..

[37] Cho Y.W., Cho Y.N., Chung S.H., Yoo G., Ko S.W. (1999). Water-soluble chitin as a wound healing accelerator. Biomaterials.

[38] Dai T., Tanaka M., Huang Y.Y., Hamblin M.R. (2011). Chitosan preparations for wounds and burns: antimicrobial and wound-healing effects. Expert Rev. Anti Infect. Ther..

[39] Howling G.I., Dettmar P.W., Goddard P.A., Hampson F.C., Dornish M., Wood E.J. (2001). The effect of chitin and chitosan on the proliferation of human skin fibroblasts and keratinocytes in vitro. Biomaterials.

[40] Yoshikawa T., Reusch M., Zuerbig V., Cimalla V., Lee K.H., Kurzyp M. (2016). Electrostatic self-assembly of diamond nanoparticles onto Al-and N-polar sputtered aluminum nitride surfaces. Nanomaterials.

[41] Wehling J., Dringen R., Zare R.N., Maas M., Rezwan K. (2014). Bactericidal activity of partially oxidized nanodiamonds. ACS Nano.

[42] Benali S., Aouadi S., Dechief A.L., Murariu M., Dubois P. (2015). Key factors for tuning hydrolytic degradation of polylactide/zinc oxide nanocomposites. Nanocomposites.

[43] Xu H., Fang Z., Tian W., Wang Y., Ye Q., Zhang L. (2018). Green fabrication of amphiphilic quaternized β-chitin derivatives with excellent biocompatibility and antibacterial activities for wound healing. Adv. Mater..

[44] Li W., Lu L., Yan F., Palasantzas G., Loos K., Pei Y. (2023). High-performance triboelectric nanogenerators based on TPU/mica nanofiber with enhanced tribo-positivity. Nano Energy.

[45] Robertson J., McGoverin C., Vanholsbeeck F., Swift S. (2019). Optimisation of the protocol for the liVE/DEAD®BacLightTM bacterial viability kit for rapid determination of bacterial load. Front. Microbiol..

[46] Zachari M.A., Chondrou P.S., Pouliliou S.E., Mitrakas A.G., Abatzoglou I., Zois C.E. (2014). Evaluation of the alamarblue assay for adherent cell irradiation experiments. Dose Response.

[47] Villaschi S., Nicosia R.F. (1994).

[48] Morimune-Moriya S., Yada S., Kuroki N., Ito S., Hashimoto T., Nishino T. (2020). Strong reinforcement effects of nanodiamond on mechanical and thermal properties of polyamide 66. Compos. Sci. Technol..

[49] Di Y., Iannace S., Di Maio E., Nicolais L. (2005). Poly(lactic acid)/organoclay nanocomposites: thermal, rheological properties and foam processing. J. Polym. Sci. B Polym. Phys..

[50] Chen Q., Wu Y., Pu Y., Zheng Z., Shi C., Huang X. (2010). Synthesis and characterization of quaternized β-chitin. Carbohydr. Res..

[51] Hong Y., Brown D.G. (2008). Electrostatic behavior of the charge-regulated bacterial cell surface. Langmuir.

[52] Metwally S., Stachewicz U. (2019). Surface potential and charges impact on cell responses on biomaterials interfaces for medical applications. Mater. Sci. Eng. C.

[53] Zhang J., Coote M.L., Ciampi S. (2021). Electrostatics and electrochemistry: mechanism and scope of charge-transfer reactions on the surface of tribocharged insulators. J. Am. Chem. Soc..

[54] Zhu L., Wang Q. (2012). Novel ferroelectric polymers for high energy density and low loss dielectrics. Macromolecules.

[55] Cao K., Zhang F., Zaeri A., Zgeib R., Chang R.C. (2022). Quantitative investigation into the design and process parametric effects on the fiber-entrapped residual charge for a polymer melt electrohydrodynamic printing process. Macromol. Mater. Eng..

[56] Raja I.S., Lee S.H., Kang M.S., Hyon S.H., Selvaraj A.R., Prabakar K. (2022). The predominant factor influencing cellular behavior on electrospun nanofibrous scaffolds: wettability or surface morphology?. Mater. Des..

[57] Xie F., Bian X., Lu Y., Xia T., Xu D., Wang Y. (2022). Versatile antibacterial surface with amphiphilic quaternized chitin-based derivatives for catheter associated infection prevention. Carbohydr. Polym..

[58] Arima Y., Iwata H. (2007). Effect of wettability and surface functional groups on protein adsorption and cell adhesion using well-defined mixed self-assembled monolayers. Biomaterials.

[59] Gao L., Li M., Ehrmann S., Tu Z., Haag R. (2019). Positively charged nanoaggregates based on zwitterionic pillar[5]arene that combat planktonic bacteria and disrupt biofilms. Angew. Chem. Int. Ed..

[60] Xue Y., Xiao H., Zhang Y. (2015). Antimicrobial polymeric materials with quaternary ammonium and phosphonium salts. Int. J. Mol. Sci..

[61] Dominguez R., Holmes K.C. (2011). Actin structure and function. Annu. Rev. Biophys..

[62] Pollard T.D., Cooper J.A. (1979). Actin, a central player in cell shape and movement. Science.

[63] Hayakawa K., Tatsumi H., Sokabe M. (2012). Mechano-sensing by actin filaments and focal adhesion proteins. Commun. Integr. Biol..

[64] Geiger B., Bershadsky A., Pankov R., Yamada K.M. (2001). Transmembrane crosstalk between the extracellular matrix and the cytoskeleton. Nat. Rev. Mol. Cell Biol..

[65] Horzum U., Ozdil B., Pesen-Okvur D. (2014). Step-by-step quantitative analysis of focal adhesions. MethodsX.

[66] Grausova L., Bacakova L., Kromka A., Potocky S., Vanecek M., Nesladek M. (2009). Nanodiamond as promising material for bone tissue engineering. J. Nanosci. Nanotechnol..

[67] Steinerova M., Matejka R., Stepanovska J., Filova E., Stankova L., Rysova M. (2021). Human osteoblast-like SAOS-2 cells on submicron-scale fibers coated with nanocrystalline diamond films. Mater. Sci. Eng. C.

[68] Ng K.W., Khor H.L., Hutmacher D.W. (2004). In vitro characterization of natural and synthetic dermal matrices cultured with human dermal fibroblasts. Biomaterials.

[69] Tomasek J.J., Gabbiani G., Hinz B., Chaponnier C., Brown R.A. (2002). Myofibroblasts and mechano: regulation of connective tissue remodelling. Nat. Rev. Mol. Cell Biol..

[70] Jonsson K., Jensen J.A., Goodson W.H., Scheuenstuhl H., West J., Hopf H.W. (1991). Tissue oxygenation, anemia, and perfusion in relation to wound healing in surgical patients. Ann. Surg..

[71] Jonsson K., Jensen J.A., Goodson W.H., Scheuenstuhl H., West J., Hopf H.W. (1991). Tissue oxygenation, anemia, and perfusion in relation to wound healing in surgical patients. Ann. Surg..

[72] Dhivya S., Padma V.V., Santhini E. (2015). Wound dressings - a review. Biomedicine.

[73] Renuka R.R., Julius A., Yoganandham S.T., Umapathy D., Ramadoss R., Samrot A.V. (2023). Diverse nanocomposites as a potential dressing for diabetic wound healing. Front. Endocrinol..

