# Supplementary material for: Melt electrowritten poly-lactic acid /nanodiamond scaffolds towards wound-healing patches
Source: Mater Today Bio. 2024 May 31;26:101112. doi: 10.1016/j.mtbio.2024.101112 (PMC11170272; doi:10.1016/j.mtbio.2024.101112)
Supplement: Multimedia component 1 [file mmc1.docx]

**Melt electrowritten poly-lactic acid /nanodiamond scaffolds towards wound-healing patches**

Xixi Wu^a, b^, Wenjian Li^c^, Lara Herlah^a^, Marcus Koch^d^, Hui Wang^e^, Romana Schirhagl^a, *^, Małgorzata K. Włodarczyk-Biegun^b, f, *^

^a^ Department of Biomedical Engineering, University Medical Centre Groningen, Ant. Deusinglaan 1, 9713 AW Groningen, The Netherlands

^b^ Polymer Science, Zernike Institute for Advanced Materials, Faculty of Science and Engineering, University of Groningen, Nijenborgh 4, 9747 AG, The Netherlands

^c^ Advanced Production Engineering, Engineering and Technology Institute of Groningen, Faculty of Science and Engineering, University of Groningen, Nijenborgh 4, 9747 AG, The Netherlands

^d^ INM – Leibniz Institute for New Materials, Campus D2 2, 66123 Saarbrücken, Germany

^e^ Nanostructured Materials and Interfaces, Zernike Institute for Advanced Materials, Faculty of Science and Engineering, University of Groningen, Nijenborgh 4, 9747 AG, the Netherlands

^f^ Biotechnology Centre, The Silesian University of Technology, Krzywoustego 8, 44-100 Gliwice, Poland

***Corresponding authors:**

**Romana Schirhagl**

Orcid.org/0000- 3980002-8749-1054; E-mail: romana.schirhagl@gmail.com

**Małgorzata K. Włodarczyk-Biegun**

Orcid.org/0000-0003-1419-6166; E-mail: [malgorzata.wlodarczyk-biegun@polsl.pl](mailto:malgorzata.wlodarczyk-biegun@polsl.pl); m.k.wlodarczyk@rug.nl


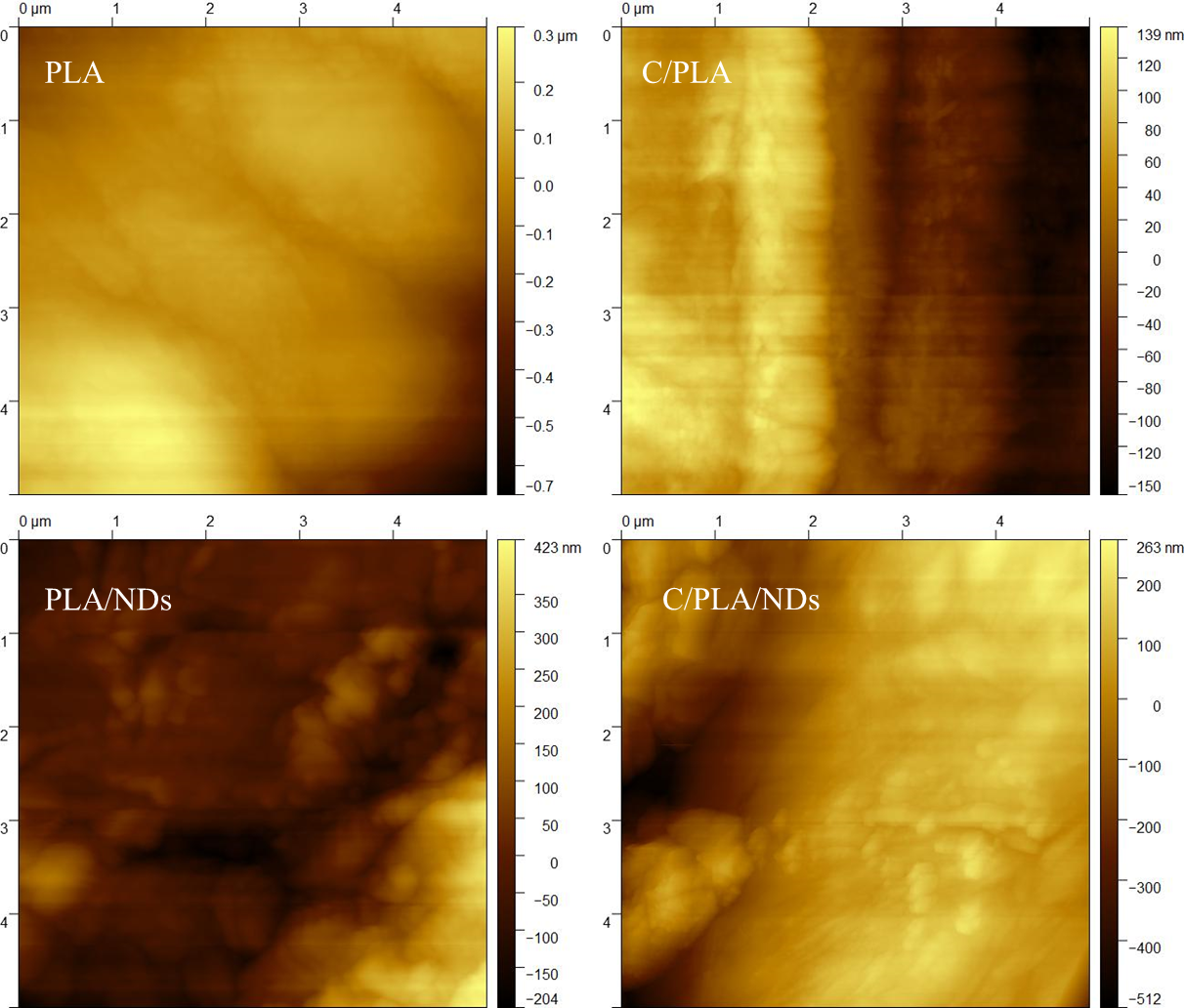


Figure S1. AFM images of PLA, C/PLA, PLA/ND, and C/PLA/ND scaffolds, obtained during the measurement of surface potential.


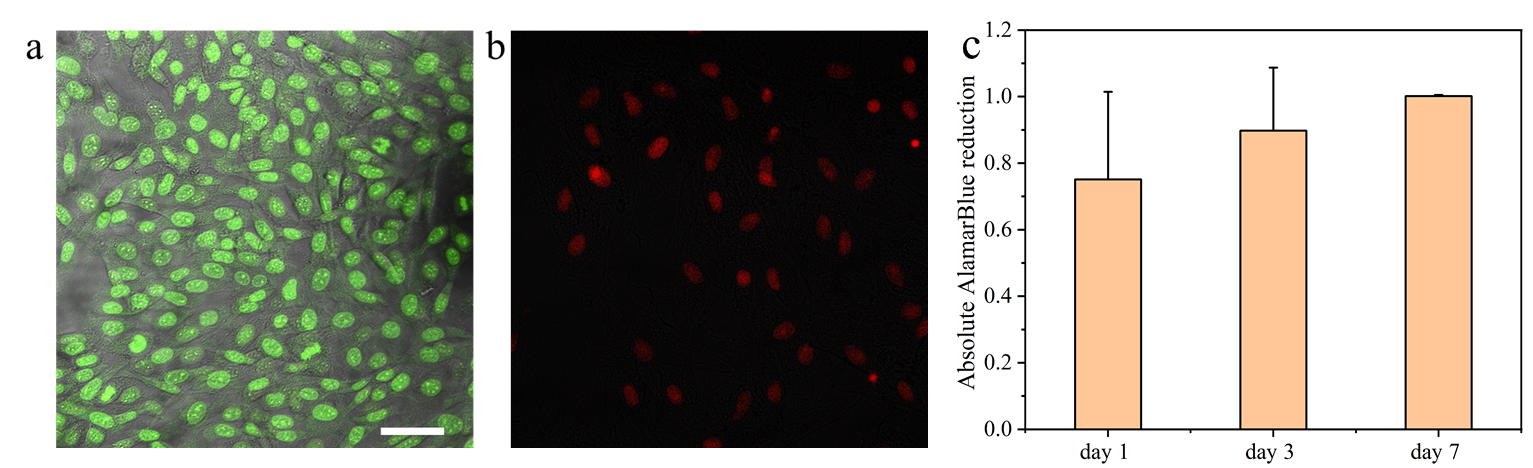


Figure S2. Viability of a) live cells ( green) and b) dead cells (red) treated with ethanol controls. scale bars are the same at 50 μm. c) Metabolic activity of NHDF cells seeded in 24-well plates during 7-day culture.


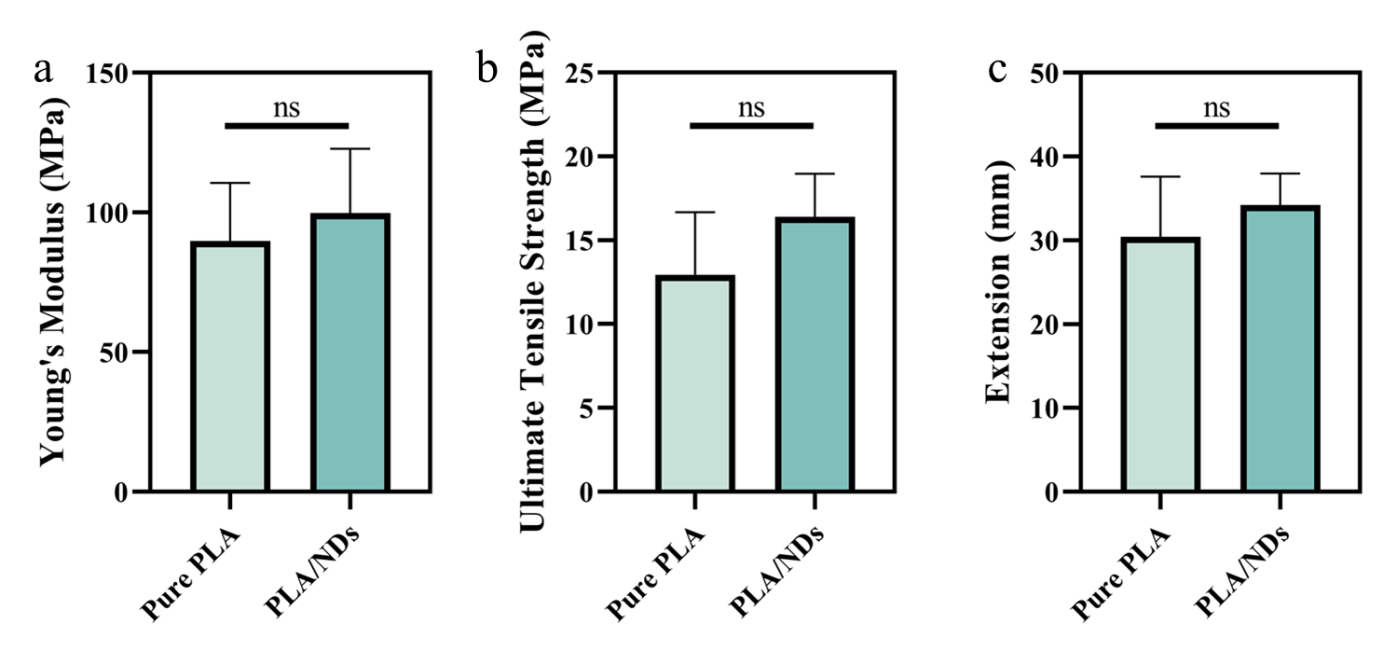


Figure S3. a) Young’s moduli b) Ultimate tensile strength (UTS) c) Maximum elongation. measured for PLA and PLA/ND films.


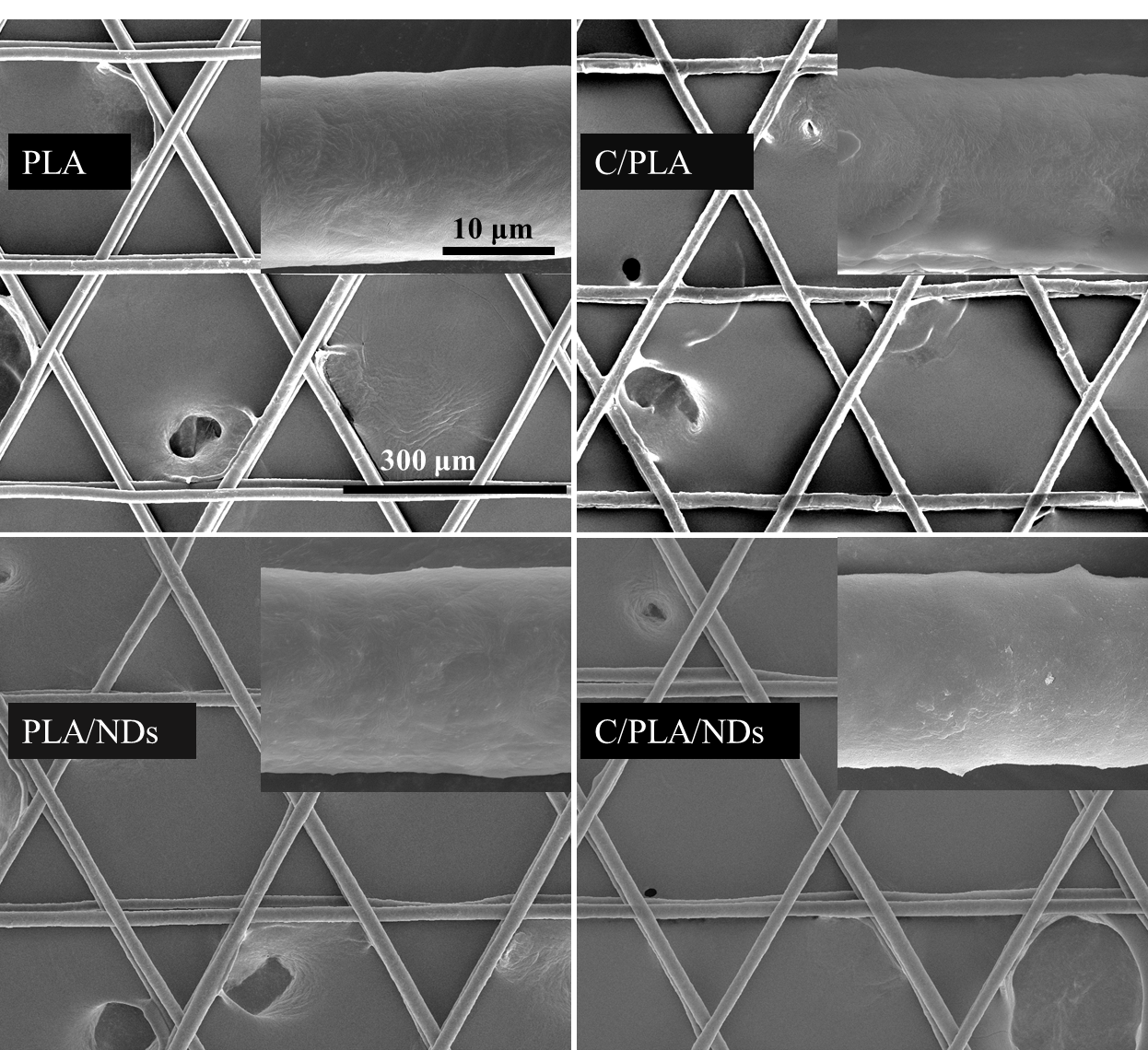


Figure S4. The morphology of the PLA-based scaffolds after 3-month degradation tests.


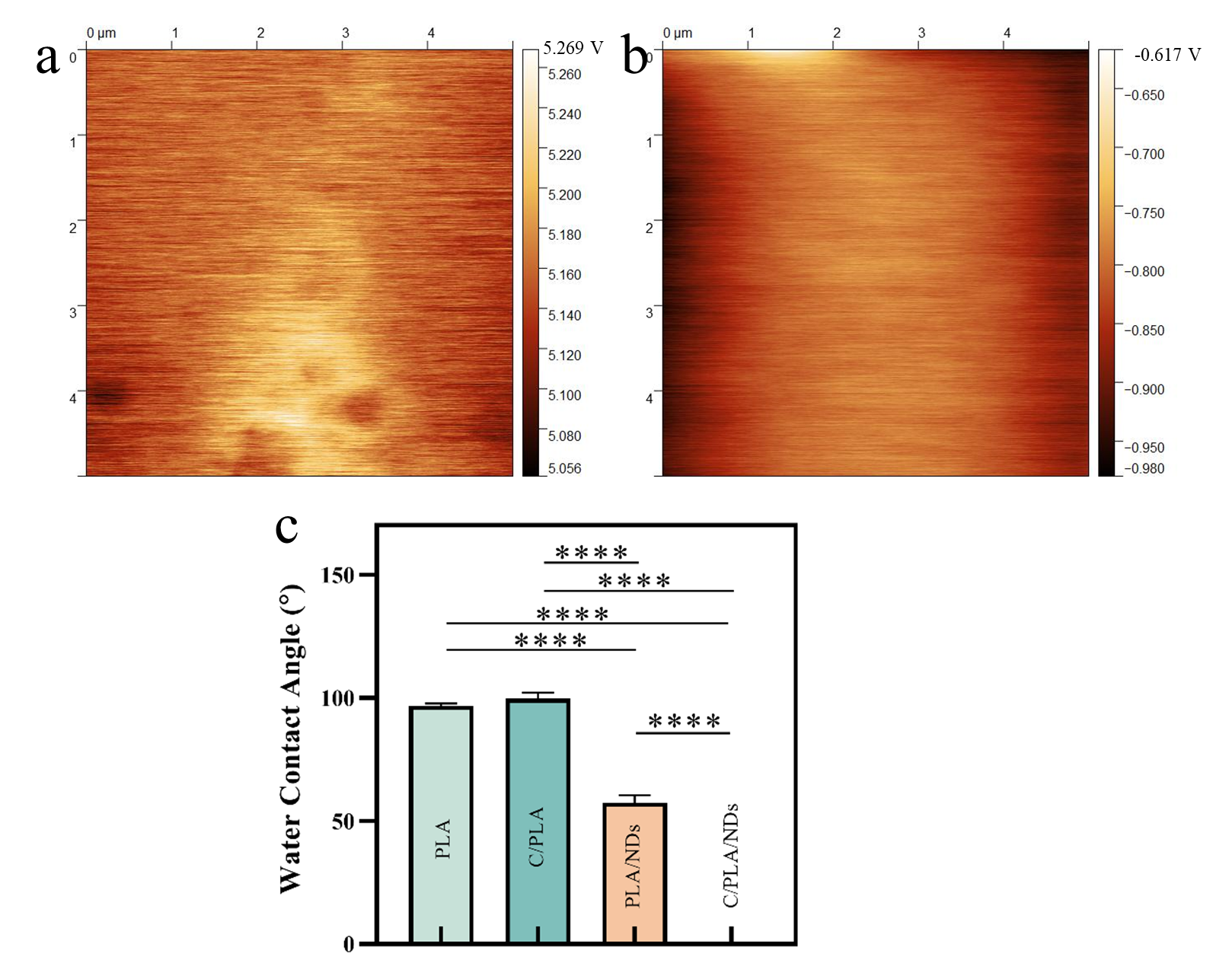


Figure S5. KPFM measurement of surface potential distribution on a) PLA melts and b) PLA/ND melt. c) Water contact angle of PLA-based films.

The release of QβC

Energy Dispersive X-Ray Analysis was used to determine the release profile of the QβC coating from PLA-based scaffolds. Samples were prepared as follows. After immersing in PBS for 5, 7 and 24 hours. PLA-based scaffolds were washes with demineralised water and mounted on a 45° pre-tilted SEM sample holder using double-sided carbon tape. Next, the samples were investigated using an FEI Quanta 400 FEG operating under high vacuum conditions at 3 kV accelerating voltage. To enhance the surface sensibility of the EDX measurements the samples were tilted to 85°. An EDAX Genesis V6.04 X-ray spectrometer measured the incoming X-rays for 200 s (3 measurements per sample). To evaluate the residual QβC coating, the peak ratio of carbon and nitrogen (exclusively in QβC) was determined by measuring the peak heights at 390 eV (nitrogen) and 270 eV (carbon). The nitrogen-carbon peak ratio of sample C/PLA/NDs was set to 100% QβC coating (=1), the peak ratio of PLA/NDs was set to 0% QβC coating (=0). For the samples without NDs the nitrogen-carbon peak ratio of pure PLA was set to 0% QβC coating, and the remaining QβC coating on C/PLA samples was determined under the assumption of an identical X-ray absorption behaviour compared to ND-containing samples.





Figure S6. The relative residual QβC on PLA and PLA/ND scaffolds after 24-h releasing in PBS.


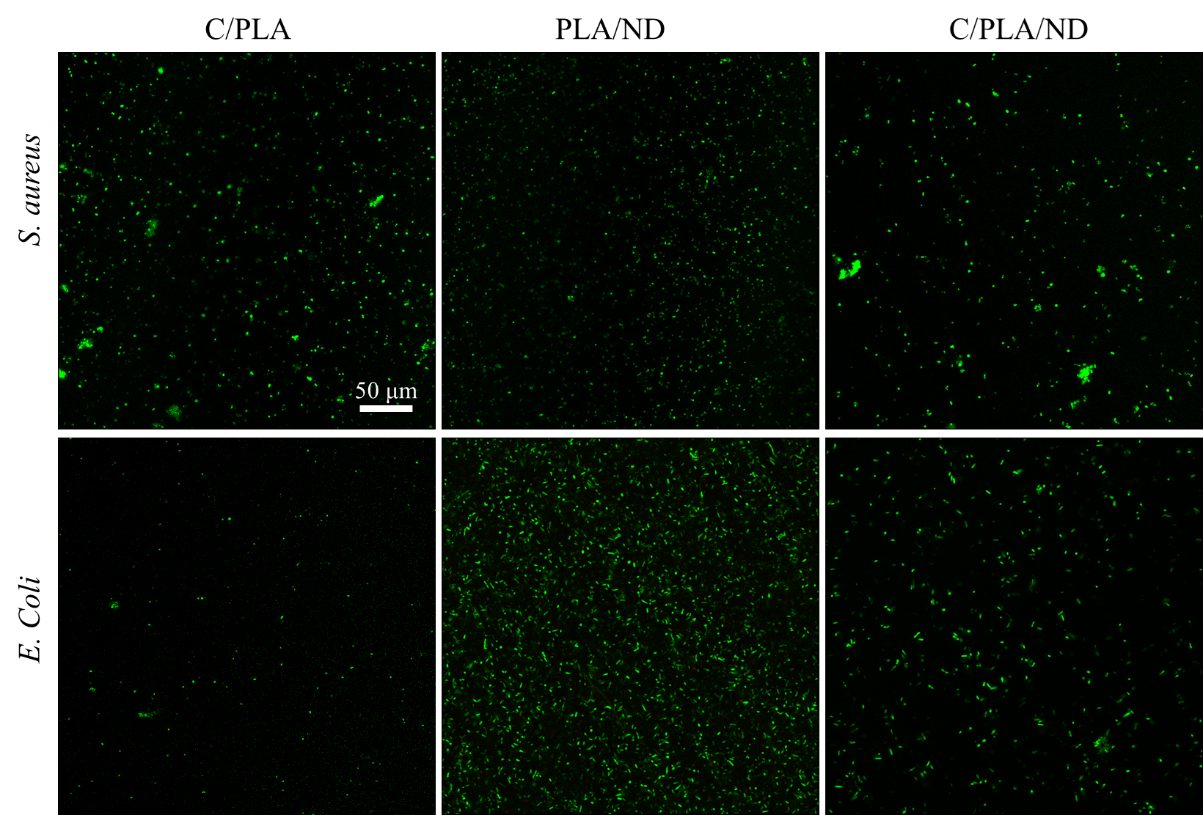


Figure S7. Live/dead staining of S. aureus and E. Coli on the scaffolds after 24-hour co-incubation.


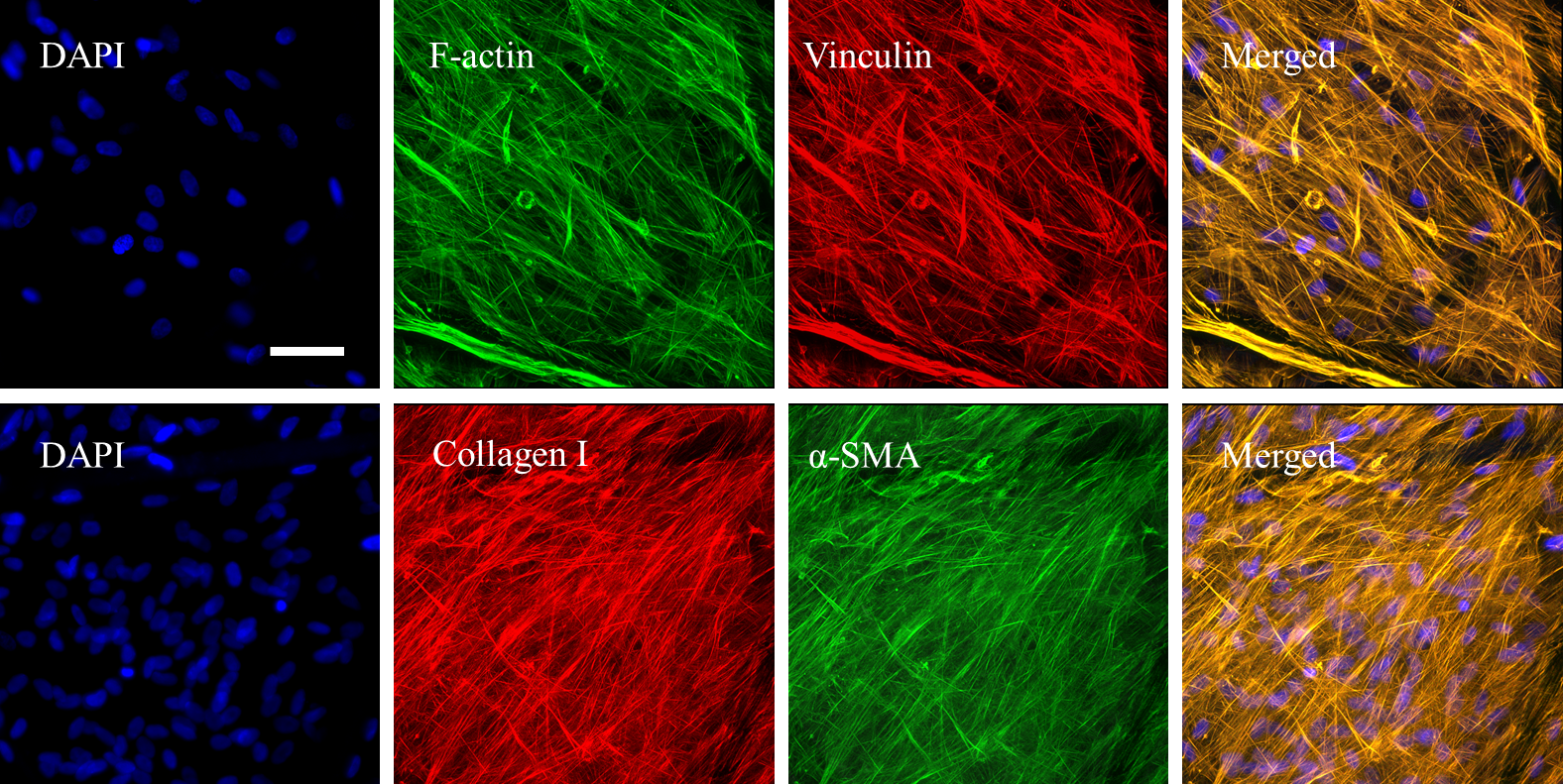


Figure S8. Immunofluorescence staining of the cells cultures in Petri dishes, e used as control groups. F-actin (in green), focal adhesive protein-vinculin (in red), Alpha -SMA (in green), collagen I (in red), and cell nuclei (in blue) were stained. The scale bar is 50 μm.


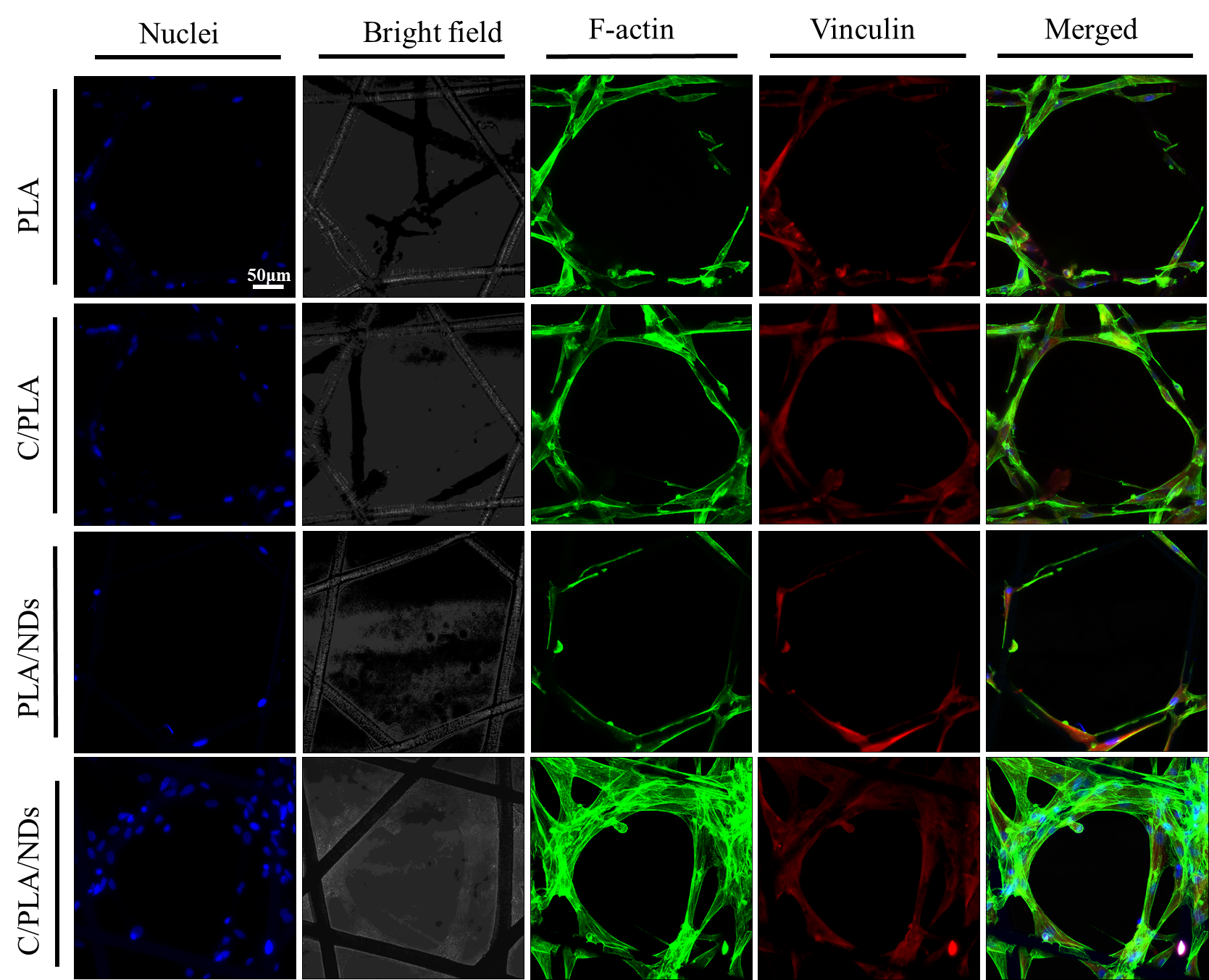


Figure S9. Cell attachment on the scaffolds on day 3. Cell nuclei (blue), F-actin (green) and focal adhesive protein-vinculin (red) were stained to show the cell adhesion on different scaffolds. Bright-field images of the scaffolds are presented with increased brightness (40%) for better visibility.


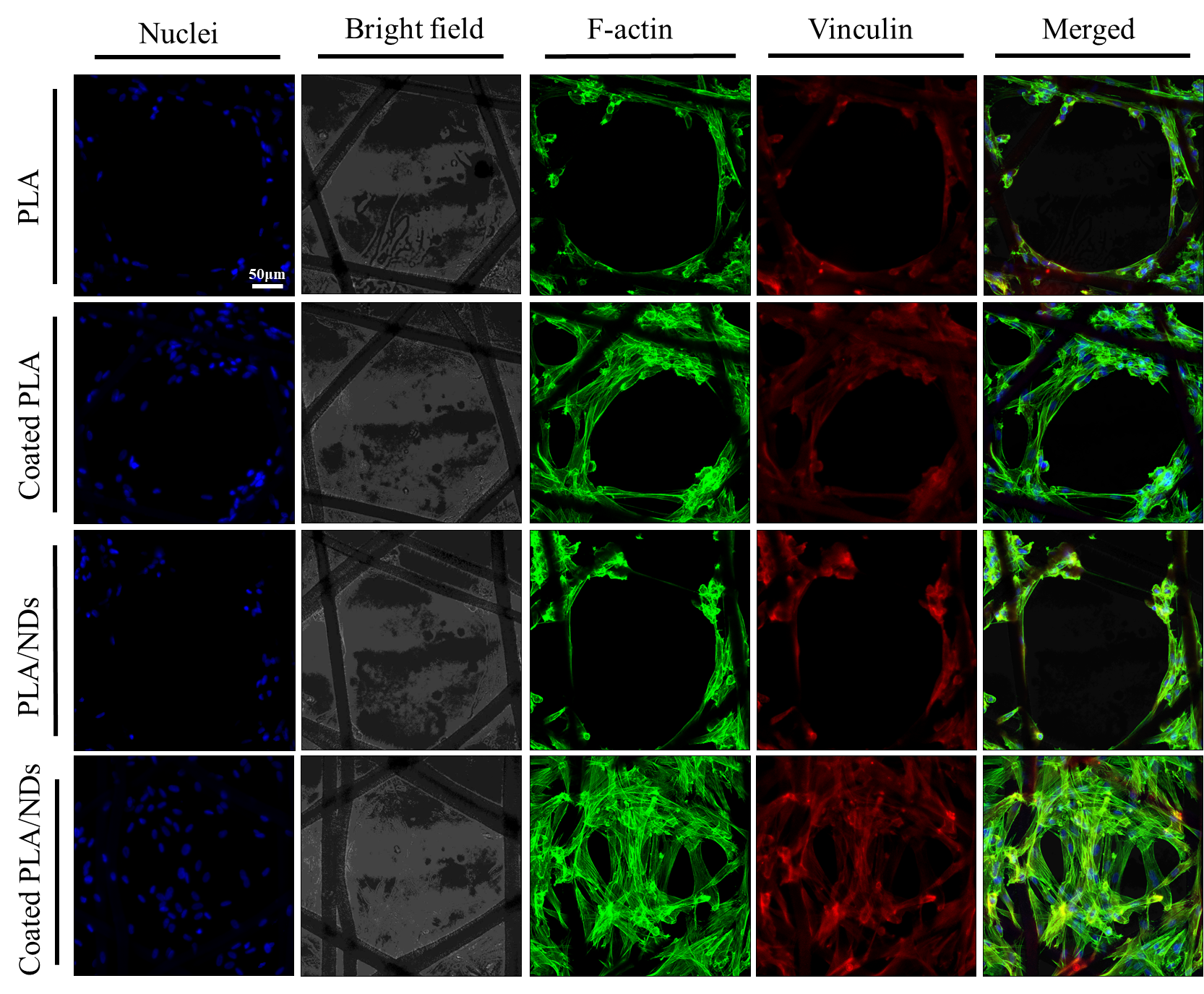


Figure S10. Cell attachment on the scaffolds at day 7. Cell nuclei (blue), F-actin (green) and focal adhesive protein-vinculin (red) were stained to show the cell adhesion on different scaffolds. Bright-field images of the scaffolds are presented with increased brightness (40%) for better visibility.


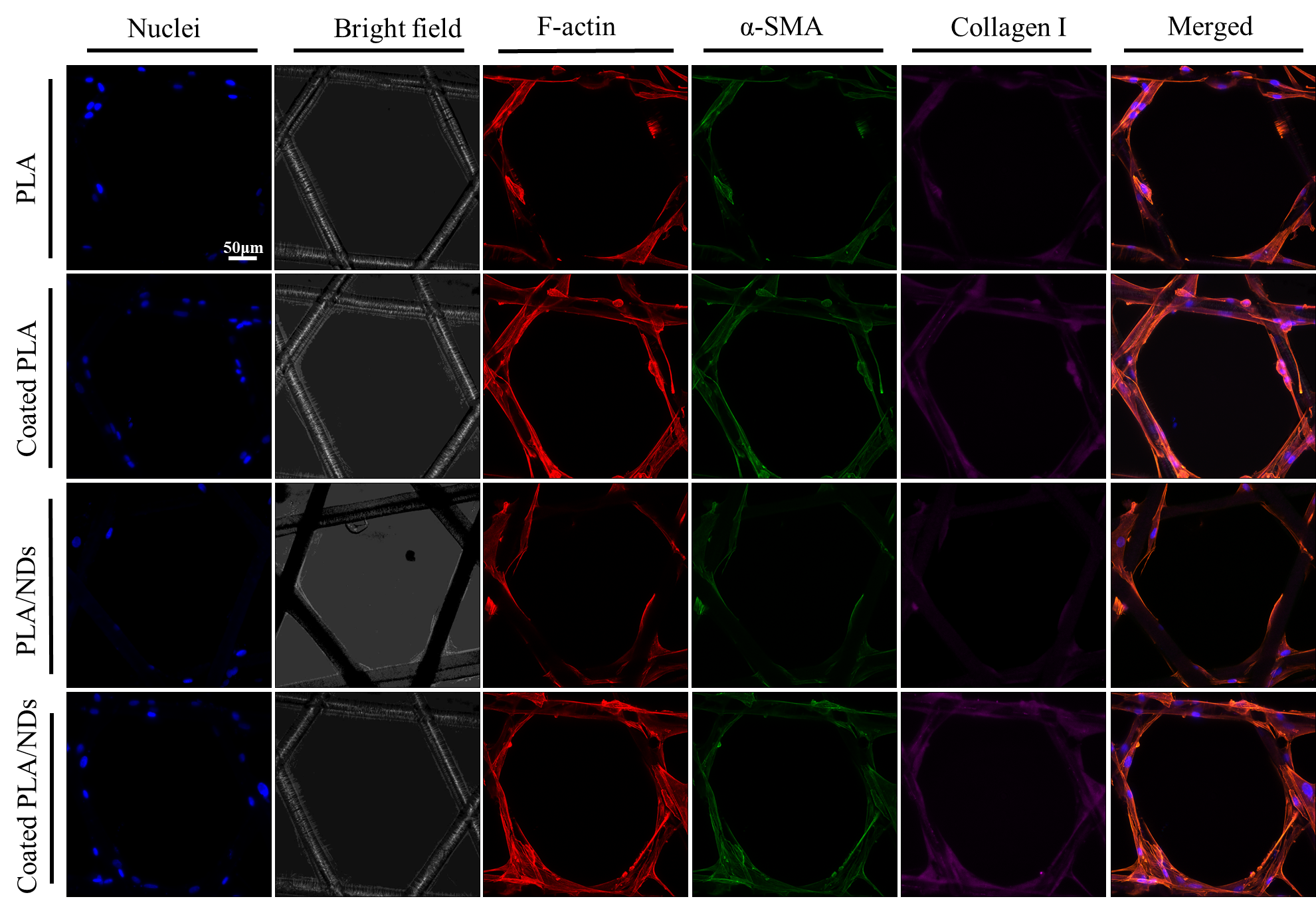


Figure S11. Expression of wound contraction protein (described by Alpha -SMA in green) and ECM remodelling protein (collagen I) on the different examined scaffolds at day 3. Bright-field images of the scaffolds are presented with increased brightness (40%) for better visibility.


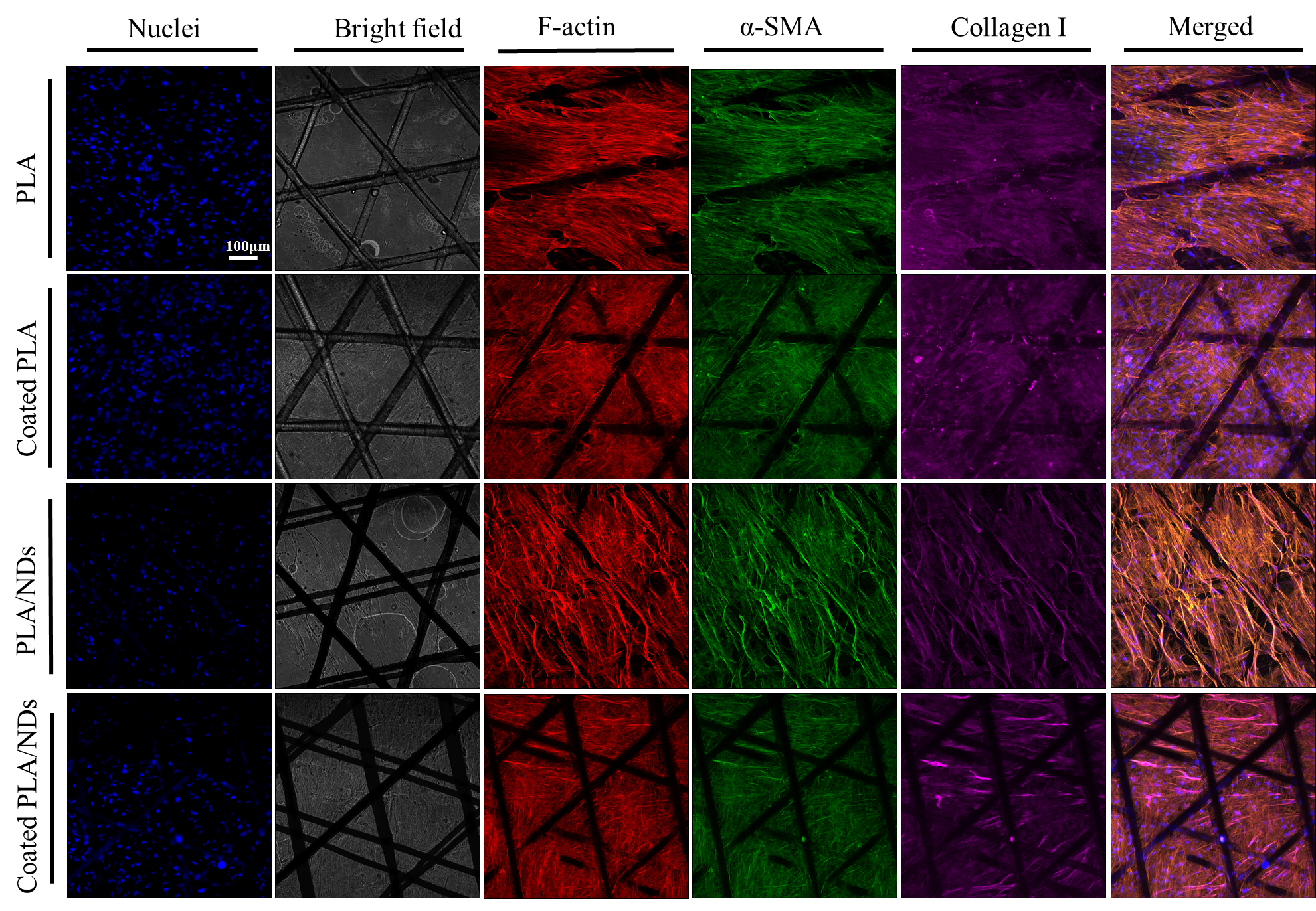


Figure S12. Expression of wound contraction protein (described by Alpha -SMA in green) and ECM remodelling protein (collagen I) on the different examined scaffolds at day 14. Bright-field images of the scaffolds are presented with increased brightness (40%) for better visibility.
